# Nanoscale biointerfaces in inter-organelle communication: membrane contact sites, organelle trafficking, and cell fate control

**DOI:** 10.3389/fcell.2026.1901757

**Published:** 2026-07-15

**Authors:** Mohamed R. Abdel-Hamed, Sameh Saber, Elsayed A. Elmorsy, Basem H. Elesawy, Maha M. Amer, Abdel-Moneim Hafez Abdel-Moneim, Alaa El-Din L. Firgany, Enas A. Mohamed, Huda Eltayeb, Ageeb M. Hassan, Mohamed El-Sayed, Ahmed Y. Kira

**Affiliations:** 1 Department of Anatomy and Histology, College of Medicine, Qassim University, Buraidah, Saudi Arabia; 2 Department of Anatomy and Embryology, Faculty of Medicine, Ain Shams University, Cairo, Egypt; 3 Department of Pharmacology, Faculty of Pharmacy, Delta University for Science and Technology, Gamasa, Egypt; 4 Department of Pharmacology and Toxicology, College of Pharmacy, Qassim University, Buraidah, Saudi Arabia; 5 Department of Clinical Pharmacology, Faculty of Medicine, Mansoura University, Mansoura, Egypt; 6 Department of Pathology, College of Medicine, Taif University, Taif, Saudi Arabia; 7 Department of Pathology, Faculty of Medicine, Mansoura University, Mansoura, Egypt; 8 Department of Physiology, College of Medicine, Qassim University, Buraidah, Saudi Arabia; 9 Department of Anatomy, Faculty of Medicine, Cairo University, Cairo, Egypt; 10 Department of Basic Sciences, College of Medicine, Princess Nourah Bint Abdulrahman University, Riyadh, Saudi Arabia; 11 Department of Pathology, College of Medicine, Qassim University, Buraidah, Saudi Arabia; 12 Faculty of Medicine, Horus University, New Damietta, Egypt; 13 Department of Pharmaceutics, Faculty of Pharmacy, Delta University for Science and Technology, Gamasa, Egypt

**Keywords:** cell fate regulation, endolysosomal trafficking, ER–mitochondria crosstalk, inter-organelle communication, membrane contact sites, organelle-targeted nanomaterials

## Abstract

Cellular and tissue organization depends on the spatial arrangement, ultrastructure, and functional coupling of organelles. This review reframes intracellular nanomaterials as nanoscale tools for interrogating and modulating membrane contact sites (MCSs), rather than simply as delivery systems. We focus on mitochondria, the endoplasmic reticulum, lysosomes, endosomes, and the nucleus because these compartments form dynamic contact networks that regulate metabolism, calcium and redox signaling, membrane trafficking, autophagy, mitophagy, chromatin organization, stress adaptation, and cell fate. Emphasis is placed on morphological and ultrastructural readouts, including mitochondrial cristae organization, fission–fusion balance, membrane-potential-dependent localization, endosomal and lysosomal trafficking, ER–mitochondria and lysosome–mitochondria communication, nuclear-pore access, chromatin organization, and inter-organelle contact-site remodeling. We discuss how particle size, surface charge, geometry, ligand presentation, and stimulus-responsive behavior influence cellular uptake, endosomal escape, organelle localization, and structural consequences within cells and tissues. A central distinction is made between intentional organelle nano-regulation, in which engineered systems are designed to engage defined subcellular mechanisms and organelle interfaces, and incidental stress responses, in which altered morphology or gene expression reflects oxidative, lysosomal, mitochondrial, inflammatory, or genotoxic injury. By organizing current evidence around MCS biology, subcellular compartmentalization, membrane trafficking, organelle dynamics, and tissue-relevant cell fate decisions, this review provides a morphology-centered framework for evaluating intracellular nanomaterials in health, disease, stem-cell biology, and regenerative bioengineering.

## 1 Introduction

Nanotechnology has evolved from focusing on extracellular control to targeting intracellular compartments, with nanomaterials now being designed to directly influence organelle function and cellular fate (McMurray et al., 2011; Zhang et al., 2021). In this stage, nanoscale topography and surface chemistry were used to regulate receptor clustering, mechanotransduction, and endocytosis (Zhang et al., 2019; Li et al., 2021). Although these approaches provided additional tools to influence cell fate, they cannot fully define the conditions governing cellular decisions. This limitation arises because many key regulatory pathways operate inside the cell, within intracellular microdomains that control metabolism, redox balance, protein activity, and gene expression in precise spatial and temporal patterns (Iqbal and Eftekharpour, 2017; Lennicke and Cochemé, 2021). This transition represents a broader development in cell biology that is increasingly treating the cell not just as a homogeneous cytosol, but rather as networks of organelles that communicate with one another. The identity of organelles remains sustained through directional transport processes, local physicochemical properties of the organelle (e.g., pH, membrane potential, ionic gradients), and through dedicated signaling routes that connect organelle state with nuclear transcriptional programs (Cohen et al., 2018; Shai et al., 2018).

Membrane contact sites (MCSs) provide a structural basis for this systems-level organization. MCSs are dynamic regions in which the membranes of two or more organelles are maintained in close apposition without complete membrane fusion, allowing localized exchange of ions, lipids, metabolites, and regulatory signals (Scorrano et al., 2019). These interfaces should be distinguished from general organelle crosstalk, which may also occur through vesicular transport or diffusible cytosolic mediators without direct membrane apposition. The formation, maintenance, and dissolution of MCSs are controlled by tethering and untethering proteins, membrane lipid composition, organelle motility, and local signaling conditions. As a result, MCS remodeling can influence organelle function and cell fate by altering the spatial efficiency of intracellular communication (Voeltz et al., 2024).

Major intracellular interfaces include ER–mitochondria, mitochondria–lysosome, and ER–endosome/lysosome contact sites. Together, these contacts coordinate Ca^2+^ and lipid exchange, organelle positioning and division, endolysosomal maturation, mitochondrial quality control, membrane repair, and stress adaptation (Sassano et al., 2022; Dematteis et al., 2024). Mitochondria–lysosome contacts contribute to mitochondrial division, ion exchange, mitochondrial quality control, and mitophagy (Wong et al., 2018), whereas ER–endolysosomal contacts regulate organelle positioning, endosomal maturation, cholesterol and phospholipid transfer, and lysosomal membrane repair (Radulovic et al., 2022). Higher-order contacts involving three or more organelles further indicate that intracellular communication is organized as a dynamic network rather than as independent binary pathways (Boutry and Kim, 2021). These contact-site functions provide the biological framework through which nanomaterial-induced alterations in organelle morphology, trafficking, and stress signaling should be interpreted.

Cell fate decisions: survival or apoptosis, quiescence or proliferation, and differentiation or self-renewal, are more frequently being recognized as emergent properties of integrated metabolic and transcriptional control processes, with the mitochondria positioned at the center of this integration (Chandel et al., 2016). Mitochondria have functions that go beyond ATP production; they also mediate redox signaling, support apoptosis, and undergo dynamic remodeling which ultimately alters their bioenergetic and signaling capabilities (Rambold and Pearce, 2018). The correlation between metabolism at the mitochondria and developmental progression and lineage determination (e.g., stem and progenitor cells) has been documented extensively where metabolic remodeling occurs concurrently with epigenetic reprogramming and transcriptional circuitry, thereby influencing cellular identity and plasticity (Carey et al., 2015; Wanet et al., 2015; Labusca and Zara-Danceanu, 2025). Throughout this review, we distinguish between intentional regulation, where nanomaterials are engineered to deliver specific effectors or engage defined intracellular pathways and incidental regulation, where gene expression shifts arise secondarily from stress responses (e.g., oxidative stress, lysosomal dysfunction, or genotoxic signaling). This distinction is critical for interpreting ‘cell identity’ outcomes and for translating organelle-targeted systems toward safe, controllable interventions.

Mitochondria communicate with the nucleus to regulate gene expression based on metabolic status. Nanomaterials targeting mitochondrial function can influence this retrograde signaling, thereby modulating nuclear gene activity and cell fate decisions (Walker et al., 2025). This mitonuclear communication has been an integral principle in the understanding of both genetics and metabolism for decades, providing the mechanistic link between mitochondrial state and nuclear transcriptional regulation (Guha and Avadhani, 2013).

In parallel, lysosomes and the autophagy–lysosome system regulate cellular quality control and energetic homeostasis by controlling turnover of proteins and organelles, including selective removal of damaged mitochondria (mitophagy) (Narendra et al., 2008). Lysosomes serve as a critical decision point for nanomaterials, determining whether they are degraded, sequestered, or released into the cytosol. Their role in autophagy and mitophagy is vital for controlling cellular stress and fate determination (Wang et al., 2018). The connection between lysosomal disruption and autophagy dysfunction to nanomaterial—cell interactions has been well established in the mechanistic toxicity literature and provides further evidence that lysosomes are both barriers and hubs that can re-direct signaling and/or fate determination (Lunov et al., 2011; Xu et al., 2016).

Although nanoscale control of cell-surface signaling can regulate receptor organization, mechanotransduction, and cellular uptake, it does not provide direct control over the intracellular interfaces that determine metabolic thresholds, calcium signaling, redox adaptation, membrane homeostasis, and stress responses. Organelle-targeted nanomaterials may address this limitation by enabling localized perturbation of intracellular biochemistry and, when appropriately designed, the molecular processes operating at MCSs (Marrache and Dhar, 2012). For mitochondria, targeting strategies such as lipophilic cations (notably triphenylphosphonium-based motifs) exploit the large negative membrane potential across the inner mitochondrial membrane to drive accumulation (Zielonka et al., 2017). For nuclear targeting, entry into the nucleus is controlled by the nuclear envelope and nuclear pore complexes, favoring strategies that deploy nuclear localization signals, DNA nanostructures, or assemblies that orchestrate uptake, endosomal escape, and import (Pan et al., 2012). Nevertheless, localization within a single organelle does not demonstrate MCS engagement; direct modulation of an inter-organelle interface requires additional structural and functional validation.

While recent reviews on organelle-targeted nanomaterials have documented the rapid expansion of strategies for targeting mitochondria, nuclei, and lysosomes, these works are predominantly organized around application-oriented delivery strategies (Buck et al., 2025; Gao L. et al., 2025; Soukar et al., 2025). In contrast, the present review is structured around MCSs as nanoscale intracellular control hubs that coordinate organelle architecture, molecular exchange, stress adaptation, and cell fate independently of a specific pathological context. The review critically identifies current knowledge gaps, including the limited investigation of defined multi-organelle contact networks, insufficient understanding of the long-term consequences of nano-induced MCS remodeling, and the difficulty of achieving MCS-specific precision. By outlining emerging directions such as programmable nanomaterials, systems-level approaches, and artificial organelle engineering, this work provides a forward-looking framework for MCS-directed intracellular nano-regulation in cellular and tissue biology. Accordingly, the review first introduces the structural and functional principles of MCS biology, followed by the intracellular processes that determine nanomaterial access to these interfaces. Subsequent sections examine major contact-site networks, including ER–mitochondria, mitochondria–lysosome, and ER–endolysosomal interfaces, their connections to nuclear signaling and cell fate, and the experimental requirements for demonstrating direct nano–MCS modulation.

For clarity, throughout the review, “nanomaterial” is used for broad conceptual discussions, “nanoparticle” for defined particulate systems, and “nanocarrier” when cargo transport or delivery is specifically involved.

## 2 Fundamentals of MCS biology

### 2.1 Structural definition and operational criteria of MCSs

MCSs are specialized regions in which the membranes of two or more organelles are maintained in close apposition without undergoing complete fusion. These interfaces create spatially restricted microdomains that permit rapid exchange of ions, lipids, metabolites, and regulatory signals while preserving the structural identity of the participating organelles. MCS-mediated communication therefore differs from vesicular trafficking, which depends on membrane budding, transport, docking, and fusion, and from indirect organelle crosstalk mediated solely by diffusible cytosolic factors.

The dimensions of MCSs vary according to the organelles involved and the molecular machinery operating at the interface. Most characterized contacts occur across nanoscale intermembrane distances that allow tethering proteins, ion channels, lipid-transfer proteins, and signaling complexes to interact across opposing membranes (de La Mora et al., 2021). Contact geometry is functionally important because excessive widening may prevent molecular bridging, whereas excessive narrowing may disrupt the organization of transport or signaling complexes (Dematteis et al., 2024). Experimental modulation of ER–mitochondria spacing has shown that an intermembrane distance of approximately 20 nm supports efficient mitochondrial Ca^2+^ uptake and oxidative metabolism, demonstrating that MCS function depends on contact architecture rather than proximity alone (Dematteis et al., 2024).

A functional MCS should be distinguished from incidental organelle colocalization. At minimum, the interface should show reproducible close membrane apposition, preservation of separate membrane identities, regulated formation or dissolution, and association with a measurable biological process. Such processes may include calcium transfer, non-vesicular lipid exchange, organelle positioning, mitochondrial division, lysosomal membrane repair, autophagy, mitophagy, metabolic adaptation, or stress signaling (Scorrano et al., 2019). Apparent overlap between organelle markers in conventional fluorescence microscopy is insufficient because limited spatial resolution, perinuclear crowding, or changes in organelle size may create false impressions of direct contact (Dunn et al., 2011).

MCSs are dynamic structures that can form, expand, redistribute, or dissolve in response to metabolic demand, nutrient availability, calcium signals, oxidative stress, membrane injury, or cell-fate transitions (Voeltz et al., 2024). Accordingly, MCS remodeling may involve changes in contact number, length, intermembrane distance, duration, subcellular distribution, or molecular composition (Giamogante et al., 2020). These variables are not necessarily equivalent; an increase in contact abundance, for example, does not establish enhanced function if the spacing or protein composition is unsuitable for molecular exchange (Dematteis et al., 2024). Structural observations should therefore be interpreted together with contact-dependent functional measurements.

### 2.2 Molecular regulation and principal functions of membrane contact sites

MCS formation and dissolution are controlled by coordinated interactions among structural tethers, membrane adaptors, lipid-transfer proteins, ion channels, small GTPases, cytoskeletal regulators, and signaling enzymes. No single tethering mechanism is shared by all organelle pairs. Instead, each interface contains a specific molecular assembly that reflects the organelles involved and the physiological function performed at the contact.

Structural tethers either bridge opposing membranes directly or recruit additional proteins that stabilize membrane proximity. At ER–mitochondria contacts, representative regulators include the VAPB–RMDN3/PTPIP51 complex and PDZD8-associated assemblies (Stoica et al., 2014; Hirabayashi et al., 2017). At ER–endolysosomal contacts, VAP proteins interact with Rab7-associated factors and lipid-transfer proteins such as ORP1L (Wijdeven et al., 2016). Mitochondria–lysosome contacts are regulated partly by Rab7 activity, whereas TBC1D15-dependent Rab7 hydrolysis contributes to contact untethering (Wong et al., 2018). These examples demonstrate that contact formation and dissolution are actively controlled rather than resulting from random organelle collision.

Several MCS proteins perform both structural and transport functions. Lipid-transfer proteins can bind opposing membranes while transferring cholesterol, phosphatidylserine, phosphoinositides, or other lipids through hydrophobic domains (Chung et al., 2015). Similarly, ion channels and associated scaffolds organize localized calcium-transfer microdomains (Katona et al., 2022). Consequently, changing the abundance, localization, or activity of an MCS protein may alter both membrane proximity and the molecular process conducted at the interface.

Membrane lipid composition also influences MCS assembly by regulating membrane charge, curvature, protein recruitment, and binding of lipid-sensing domains. Phosphoinositides, cholesterol, and phosphatidylserine are particularly important in defining organelle identity and recruiting contact-site machinery (Rocha et al., 2009; Chung et al., 2015). In parallel, microtubule- and actin-dependent organelle movement determines where and when membranes encounter one another. Cytoskeletal transport therefore links MCS formation to broader processes of organelle positioning, intracellular trafficking, and membrane remodeling (Korobova et al., 2013; Raiborg et al., 2015).

The major functions of MCSs include calcium exchange, non-vesicular lipid transport, metabolic regulation, organelle division, membrane repair, autophagy, mitophagy, and stress adaptation. ER–mitochondria contacts coordinate calcium transfer, phospholipid metabolism, mitochondrial fission, bioenergetics, and apoptotic signaling (Carpio et al., 2021; Katona et al., 2022). Mitochondria–lysosome contacts regulate mitochondrial division, ion handling, and quality control, whereas ER–endosome and ER–lysosome contacts contribute to organelle positioning, maturation, cholesterol transport, and lysosomal membrane repair (Raiborg et al., 2015; Höglinger et al., 2019; Giamogante et al., 2020). Higher-order contacts involving three or more organelles further integrate these processes into coordinated intracellular networks.

MCS function is therefore determined by a balance between contact formation and contact dissolution. Insufficient tethering may reduce molecular exchange or impair organelle coordination, whereas excessive or prolonged contact may promote calcium overload, abnormal lipid redistribution, altered organelle motility, or defective autophagic flux. Evaluation of MCS biology should consequently consider both loss and gain of contact as potentially disruptive, depending on the biological context.

### 2.3 Relevance of membrane contact sites to nanomaterial interactions

For intracellular nanomaterials, cellular uptake and organelle localization represent prerequisites for MCS engagement but do not demonstrate direct modulation of a contact site. Most nanomaterials enter cells through endocytic pathways and are subsequently trafficked through early endosomes, late endosomes, and lysosomes (Behzadi et al., 2017). Endosomal entrapment, recycling, degradation, or lysosomal sequestration can prevent intact nanomaterials or their released cargoes from reaching cytosolic organelle surfaces and inter-organelle interfaces (Peñaloza et al., 2017).

Even when a nanomaterial colocalizes with an individual organelle, the signal may reflect vesicular trapping, perinuclear accumulation, membrane association, or localization of the released cargo rather than the intact carrier (Tammam et al., 2017; Johansson et al., 2025). Direct nano–MCS engagement therefore requires evidence that the nanomaterial, its surface components, or its cargo reaches a defined inter-organelle interface and alters a structural or functional property of that contact.

Nanomaterials may influence MCSs through several mechanisms. They may alter membrane lipid composition, local calcium gradients, redox status, membrane curvature, organelle motility, tether recruitment, or post-translational regulation of contact-site proteins (Dey et al., 2022; Luo and Wang, 2023). These effects may stabilize, disrupt, or reprogram MCSs depending on particle size, surface chemistry, intracellular dose, and exposure conditions (Jin et al., 2021). However, similar changes may also occur indirectly as consequences of oxidative stress, lysosomal membrane damage, mitochondrial dysfunction, inflammation, or genotoxicity.

A rigorous nano–MCS analysis should therefore distinguish four levels of evidence: cellular uptake, organelle localization, physical association with a contact site, and functional modulation of contact-dependent molecular exchange or signaling. Claims of direct MCS regulation should ideally be supported by quantitative changes in contact abundance, length, spacing, duration, or molecular composition, together with assessment of the relevant transferred ion, lipid, metabolite, or downstream signaling pathway. Perturbation or rescue of the proposed contact-site machinery provides additional evidence that the observed biological outcome is MCS-dependent.

**FIGURE 1:**
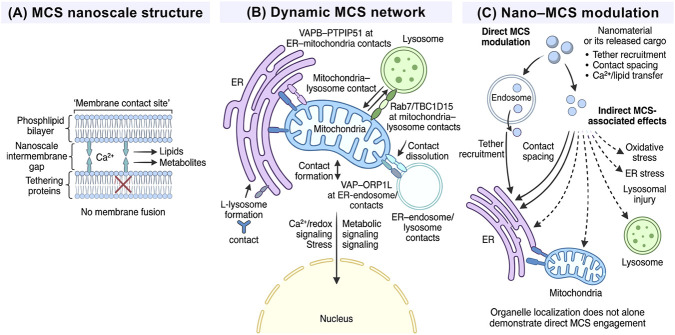
Nanoscale architecture, dynamic organization, and nanomaterial modulation of membrane contact sites. **(A)** Membrane contact sites (MCSs) are nanoscale regions in which the membranes of two organelles are maintained in close apposition without membrane fusion. Tethering proteins bridge the intermembrane gap and support localized transfer of Ca^2+^, lipids, metabolites, and regulatory signals. **(B)** MCSs form a dynamic intracellular network involving the endoplasmic reticulum (ER), mitochondria, lysosomes, late endosomes, and the nucleus. Representative regulatory machinery includes VAPB–RMDN3/PTPIP51 at ER–mitochondria contacts, Rab7 and TBC1D15 at mitochondria–lysosome contacts, and VAP–ORP1L at ER–endosome/lysosome contacts. Contact formation, remodeling, and dissolution regulate molecular exchange, organelle dynamics, and downstream Ca^2+^, redox, metabolic, and stress-response signaling to the nucleus. **(C)** Nanomaterials or their released cargoes may influence MCSs directly by altering tether recruitment, contact spacing, or contact-dependent Ca^2+^ and lipid transfer. Indirect MCS-associated effects may arise through oxidative stress, ER stress, lysosomal injury, or altered intracellular trafficking. Solid arrows indicate direct or contact-associated effects, whereas dashed arrows indicate indirect stress-mediated effects. Organelle localization alone does not demonstrate direct MCS engagement. ER, endoplasmic reticulum; MCS, membrane contact site; ORP1L, oxysterol-binding protein-related protein 1L; TBC1D15, TBC1 domain family member 15; VAPB, vesicle-associated membrane protein-associated protein B; RMDN3/PTPIP51, regulator of microtubule dynamics 3/protein tyrosine phosphatase-interacting protein 51.

This framework is essential for separating intentional nano-regulation from incidental contact-site disruption. Intentional regulation refers to nanomaterials designed to engage a defined MCS mechanism, whereas incidental disruption occurs when contact remodeling arises secondarily from generalized organelle injury. Applying these criteria allows nanomaterial-induced changes in organelle morphology, trafficking, metabolism, and cell fate to be interpreted with greater mechanistic precision. Figure 1 provides an integrated overview of the nanoscale architecture of MCSs, their dynamic organization across major intracellular membrane systems, and the potential direct and indirect mechanisms through which nanomaterials may influence these interfaces.

### 2.4 Evidence linking nanomaterial architecture to inter-organelle signaling

Nanomaterial surface architecture may influence inter-organelle signaling through two mechanistically distinct routes. In the first, surface-displayed targeting units physically recruit or bridge two organelles and thereby alter a defined contact-dependent process. In the second, particle size, charge, coating, or surface reactivity determines intracellular trafficking and accumulation within one organelle, which subsequently modifies its communication with another organelle. These mechanisms should not be considered equivalent because only the former represents deliberately engineered contact-site modulation.

A direct example was provided by Xiao et al., who developed programmable DNA-based regulators containing a mitochondria-targeting triphenylphosphonium unit and a CD63-binding aptamer directed toward lysosomal membranes (Xiao et al., 2025). Stimulus-dependent exposure of the CD63 aptamer enabled the construct to associate sequentially with mitochondria and lysosomes, thereby increasing mitochondria–lysosome contacts and promoting mitochondrial fission, autophagy, and metabolic changes. This study illustrates how the spatial arrangement and conditional exposure of two organelle-recognition motifs within a nanostructure can act as a synthetic inter-organelle tether rather than merely as an organelle-targeting ligand.

Nanomaterial properties may also regulate inter-organelle communication indirectly. Wang et al., demonstrated that differently sized gold nanoparticles accumulated within lysosomes to different extents and induced size-dependent lysosomal swelling and motility changes (Wang et al., 2022). These alterations increased transient lysosome–mitochondria events and promoted reversible mitochondrial fission. In another study, superparamagnetic iron oxide nanoparticles altered ER–mitochondria communication by promoting cyclooxygenase-2 localization and its association with the IP_3_R–GRP75–VDAC1 calcium-transfer complex, resulting in disturbed Ca^2+^ homeostasis and mitochondrial apoptosis (Che et al., 2020). These examples demonstrate nanomaterial-induced modulation of inter-organelle signaling, although the effects were produced through organelle accumulation or stress rather than through a purpose-designed surface tether.

Surface charge provides a further example of how nanomaterial architecture determines the probability of organelle-network engagement. Positively and negatively functionalized CdSe/ZnS quantum dots exhibited different uptake routes and intracellular distributions: positively charged particles accumulated predominantly in lysosomes, whereas negatively charged particles localized to both lysosomes and mitochondria (Liu et al., 2025). Such charge-dependent localization may alter which inter-organelle pathways are affected, but localization to two organelles alone does not demonstrate physical contact-site engagement or contact-dependent signaling.

Collectively, these findings indicate that nanomaterial size, charge, coating, ligand identity, and spatial presentation can influence inter-organelle communication by controlling organelle localization, membrane perturbation, tether recruitment, or physical bridging. Nevertheless, direct MCS modulation should be claimed only when structural changes at a defined contact are accompanied by altered contact-dependent molecular exchange or signaling and, preferably, causal perturbation or rescue of the relevant contact-site machinery.

## 3 Intracellular journey of nanomaterials: from membrane entry to subcellular localization and morphology

**FIGURE 2:**
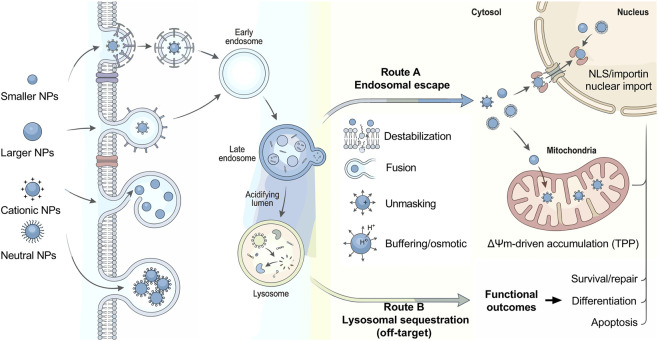
Intracellular trafficking of nanocarriers through the endosomal pathway and the endosomal escape bottleneck enabling organelle localization. Nanocarriers internalize predominantly via endocytosis and traffic through early endosomes, late endosomes, and lysosomes. Only a subset escape into the cytosol, where they may interact with mitochondria, nuclei, or other organelles. For morphology-centered interpretation, uptake should be distinguished from true organelle localization and from structural engagement. Verified targeting requires complementary localization and structural assays, because persistent vesicular trapping or perinuclear accumulation may mimic organelle targeting.

The fate of engineered nanomaterials within the cell is determined by (i) their transport across the plasma membrane; (ii) the sorting action of cellular pathways once in the endocytic network; (iii) their access to cytosolic transport mechanisms that may result in proximity to specific organelles of interest (Behzadi et al., 2017). Targeting organelles, however, is not simply about matching a single address label; it involves a cascade of interactions that determine the ultimate intracellular fate of nanomaterials (Figure 2).

### 3.1 Cellular uptake pathways and cell-type-dependent morphology

Several types of endocytosis (clathrin-mediated endocytosis, caveolae/rafts-associated pathways, macropinocytosis, phagocytosis) enable most nanomaterials to gain access to mammalian cells (Foroozandeh and Aziz, 2018). These pathways vary in terms of vesicle size, cargo sorting, maturation dynamics, and downstream trafficking, resulting in distinct probabilities for lysosomal delivery or re-cycling, transcytosis, or productive access to cytosol. Previous studies integrating nanomaterial design with endocytic cell biology emphasize that a single formulation may engage multiple routes depending on cell type, local membrane composition, corona formation, and dose (Dunn et al., 2014; Zhang et al., 2016; Cai et al., 2024).

Also, “non-endocytic” transport may occur along a range of translocation events for ultrasmall, amphipathic, and/or peptide-decorated nanomaterials, including temporary destabilization of the membrane, migration of lipids into new locations, and/or uptake facilitated by cell-penetrating peptides (CPPs) (Borrelli et al., 2018). However, even with CPPs, endosomal entrapment remains a significant challenge for ensuring that the nanomaterials reach target organelles such as the mitochondria or nucleus (Wadia et al., 2004; Madani et al., 2011; Guidotti et al., 2017).

### 3.2 Endosomal escape, intracellular trafficking, and structural validation

After particles internalization, they usually travel from early endosomes to late endosomes and lysosomes, where progressive acidification and hydrolase exposure can sequester or degrade cargos. This canonical maturation pathway is a central barrier for any mechanism-driven organelle modulation that requires cytosolic or organelle membrane access (e.g., mitochondrial outer membrane or nuclear envelope) (Smith et al., 2018). The body of literature on the nanoparticle uptake and intracellular trafficking reveals that the uptake route, size distribution, and the chemical surface characteristics are key factors in determining the ultimate fate of NPs, whether degradation, recycling compartments, or non-canonical sorting pathways (Hagedorn et al., 2024).

The term “endosomal escape” describes how nanosystems, or their cargoes, move out of their endosomes, reaching into the cytosol, or internal environment of the cell. Various mechanisms are described in the literature, which implement recurring physicochemical routes to carry out this process (Bhangu et al., 2024). Acidification can activate pH-responsive chemistries that destabilize membranes or trigger structural transitions in carriers, including membrane fusion tendencies and lipid rearrangements that favor non-bilayer intermediates (Wittrup et al., 2015). Ionizable lipid systems, in particular, are frequently discussed in terms of acid-driven lipid mixing and formation of destabilizing structures that promote release. In other platforms, escape can arise from pore formation or membrane rupture mediated by lytic motifs or cell-penetrating/endo-lytic peptides (Patel et al., 2019), as well as from osmotic stress mechanisms associated with buffering polymers; the latter has been widely discussed but remains sensitive to formulation- and context-dependent interpretation (Benjaminsen et al., 2013). External triggers, including light-activated reactive oxygen species, have also been used to induce membrane disruption and release.

However, the functional efficiency of cytosolic delivery is very heterogeneous across different vesicle subtypes at different points in time (Liu et al., 2024). Consequently, when performing validations, most methodical reviews and quantitative analyses emphasize that endosomal escape should not only be determined by the colocalization or uptake of a delivery agent, but true cytosolic release can be validated through utilizing functional assays that distinguish true cytosolic release from partial leakage, vesicle rupture artifacts, or persistent intraluminal trapping (Kilchrist et al., 2019; Beach et al., 2021).

### 3.3 Physicochemical determinants of organelle localization and ultrastructural effects

Organelle localization by engineered nanomaterials is not an intrinsic property but emerges from a sequence of physicochemical interactions that govern cellular bioprocessing, including biomolecular corona formation, membrane binding energetics, endocytic pathway selection, vesicle maturation, cytosolic access, and intracellular transport. From a cell- and tissue-biology perspective, these design variables are important because they determine not only whether a nanomaterial reaches mitochondria, nuclei, or lysosomes, but also whether it measurably alters organelle morphology, contact sites, chromatin architecture, or tissue-relevant cell behavior.

Particle size is a quantitatively validated determinant of intracellular entry route and downstream trafficking. In a standardized size-controlled experiment with spherical gold NPs, the greatest uptake was observed with ∼50 nm particles, compared to 14 or 74 nm particles, indicating a size related, non-linear relationship with uptake under identical conditions (Chithrani et al., 2006). In parallel, latex fluorescent beads were evaluated and found that the size of a particle determines which endocytic pathway will be used for cellular uptake, i.e., beads <200 nm use clathrin-coated pits and >500 nm do not, indicating that the maximum size for clathrin-mediated uptake is ≤200 nm (Rejman et al., 2004). Other systematic studies support the conclusion that size (along with other engineering parameters) affects not only the efficiency of internalization but also the subsequent trafficking of a particle. In particular, ultra-small dimensions allow access to organelles through differing subcellular distribution patterns; for example, <10 nm AuNPs entered the nucleus while 10–16 nm AuNPs could not (Huo et al., 2014).

Surface charge is another determinant of nanoparticle–cell interactions as it governs electrostatic association with the glycocalyx, membrane lipids, and adsorbed serum proteins. Multiple experimental studies demonstrate that cationic NPs exhibit higher cellular uptake than neutral or anionic counterparts (Harush-Frenkel et al., 2007). Charge-dependent intracellular fate has also been demonstrated in mammalian cells. A recent study used ZnS quantum dots with various surface charges to evaluate how surface charge affected the uptake pathways and subcellular localization. Quantum dots with a positive charge took up more efficiently through clathrin- and caveola-mediated pathways and primarily localized to lysosomes, while quantum dots with a negative charge had different pathway uptake characteristics as well as different sub-cellular distribution patterns, such as being partially localized to mitochondria and having faster exocytosis than quantum dots with positive charge (Liu et al., 2025). However, cationic particles also tend to accumulate within lysosomes and induce membrane and oxidative stress responses, as demonstrated in experimental studies of charged NPs that linked positive surface charge to lysosomal damage and intracellular toxicity (Feng et al., 2024). In order to achieve both optimal cellular uptake and improved access to the cytosol and/or organelles, experimental systems being developed include charge-reversible and pH-responsive nanomaterials allowing for initial neutral charge at time of uptake but facilitating conversion to cationic charge in an acidic endosomal environment, thereby enhancing the efficiency of delivering bioactive agents intracellularly (Tracey et al., 2023).

Particle geometry adds an energetic–kinetic constraint on membrane deformation and therefore modulates both internalization dynamics and downstream trafficking. Anisotropic particles, used in biomimetic membranes, exhibit an engulfment profile that differs depending upon their orientation to the membrane due to the combined effects of their curvature, aspect ratio, tension of the membrane, and adhesion strength. These results show that elongation can stabilize incomplete encapsulation states rather than rapid full engulfment (Van der Ham et al., 2024). Consistently, experiments comparing spherical versus ellipsoidal soft microgels on lipid membranes show that particle shape and membrane rigidity reprogram wrapping outcomes and membrane deformation, with deep-wrapped states depending on geometry and mechanical/adhesive parameters rather than volume alone (Liu et al., 2023). In living cells, inorganic structures with high aspect ratios can enter the cells via multiple uptake pathways culminating in different types of vesicles; for instance, in breast cancer cell lines, bare gold nanorods were quantitatively taken up and visualized to be located in macropinocytic and lysosomal compartments, showing that the geometry of the gold nanorods defined their associated post-endocytic fate within the endo-lysosomal system (White et al., 2022).

Another factor is surface-displayed ligands which can enable receptor-selective entry, but they do not confer deterministic organelle targeting in isolation because intracellular fate remains constrained by engaging machinery for endocytic uptake and post-endocytic sorting (in parallel). Recently established experimental studies have shown that variations in the density and nanoscale organization of the target ligand (e.g., spacing, clustering, and/or multivalency of the target ligand) act as primary control factors that determine whether the receptor’s clustering threshold or level of productive internalization will occur versus the level of surface-bound receptor. For example, systematic variation of targeting-peptide density on polymer NPs altered C-type lectin-like molecule-1 receptor-specific uptake behavior, demonstrating that “more ligand” does not necessarily translate into higher internalization and that uptake outcomes depend on quantitative presentation parameters rather than ligand identity alone (Ackun-Farmmer et al., 2020). In an immunological receptor system in which spatial control can be imposed with nanometer precision, it was found that tight spacing of ligands (e.g., ∼7 nm versus ∼17.5 nm at constant ligand count) led to increased receptor phosphorylation and a significant enhancement in the ability of FcγR-driven phagocytosis, thereby providing a direct link between nanoscale ligand clustering, uptake efficiency, and signaling-coupled internalization (Kern et al., 2021). Consistent with a mechanistic coupling between ligand multivalency and specific endocytic structures, multivalent receptor-binding NPs exhibited avidity that depended strongly on clathrin-coated pit availability/curvature matching, indicating that ligand-functionalized NPs engagement with receptors can be amplified (or weakened) by nanoscale membrane morphology that governs receptor-mediated endocytosis (Zimmer and Goepferich, 2023).

Peptide ligands such as CPP and organelle-addressing sequences can enhance cellular uptake and bias intracellular distribution, but experimental studies show that peptide decoration alone does not guarantee cytosolic or organelle access because endosomal capture remains a dominant pathway. For example, In a quantitative study of siRNA delivery using lipid nanoparticles, fluorescence microscopy and single-cell analysis revealed that although particles entered cells efficiently, the vast majority remained trapped within endosomal compartments, and only a small fraction escaped into the cytosol, demonstrating that uptake does not equate to functional cytosolic delivery (Sahay et al., 2013).

In summary, organelle localization by engineered nanomaterials depends on coordinated physicochemical interactions, including particle size, surface charge, shape, corona composition, and functional surface modification. These interactions define uptake route, endosomal escape, cytosolic availability, and the probability of true intra-organelle localization. Because morphological and ultrastructural interpretation is central to this review, localization claims should be supported by orthogonal structural evidence, not only by uptake or perinuclear fluorescence. Table 1 summarizes key factors for nanomaterial localization and highlights the structural challenges associated with precise modulation of cellular architecture.

**TABLE 1:** Key phases and physicochemical determinants of nanomaterial localization and organelle-structure readouts.

Phase	Key mechanism	Physicochemical determinants	Challenges/considerations
Cellular uptake	Endocytosis (clathrin-, caveolae-mediated), macropinocytosis, phagocytosis	Hydrodynamic size; surface charge; shape; protein corona; ligand display for cell-surface uptake receptors	Endosomal/lysosomal sequestration; strong cell-type dependence; dose- and serum-dependent variability
Non-endocytic uptake	Membrane translocation (rare), membrane destabilization, cell-penetrating peptides (CPPs)	Amphipathicity; charge distribution; membrane affinity; deformability/softness; transient pore formation propensity	Often limited to specific formulations/cell types; can increase cytotoxicity; still frequently followed by endosomal trapping
Endosomal escape	Endosomal membrane destabilization, lipid mixing/fusion, pore formation, osmotic effects (formulation-dependent)	pH-/redox-/enzyme-responsive components; fusogenic lipids/peptides; buffering capacity (not universal); surface activity	Highly variable efficiency; hard to quantify; risk of membrane damage/inflammation; escape must be demonstrated (not assumed)
Cytosolic trafficking	Diffusion and cytoskeleton-assisted transport; perinuclear accumulation; interaction with trafficking machinery	Effective hydrodynamic size and deformability; corona evolution; charge shielding; intracellular stability; release kinetics	Pathway competition (ER/lysosome routing); premature degradation; limited cytosolic residence time
Organellar targeting	Organelle-specific transport/partitioning: importin–NPC transport (nucleus); membrane potential–driven accumulation (mitochondria); endolysosomal routing/pH partitioning (lysosomes)	NLS exposure/availability and importin engagement (nucleus); lipophilic cations (e.g., TPP) or targeting sequences (mitochondria); trafficking bias + pH-dependent partitioning (lysosomes)	Achieving organelle selectivity; cargo-size limits; cell-cycle effects for nuclear access; validate true intra-organelle localization vs. perinuclear association
Post-endocytic sorting	Endosomal maturation → recycling vs. late endosome/lysosome routing	Surface chemistry/charge; ligand display affecting endosomal maturation; stability vs. degradability; trafficking kinetics	Off-target lysosomal accumulation; recycling loss; balancing stability with timely release
Particle geometry	Shape/rigidity effects on uptake and intracellular processing	Aspect ratio; rigidity (soft vs. rigid); surface curvature; aggregation tendency	Geometry-dependent uptake varies by cell type; may alter trafficking and toxicity; manufacturing consistency
Ligand functionalization	Targeting cell-surface uptake receptors and influencing intracellular sorting; conditional exposure of targeting cues after escape	Ligand density/spacing (avidity); nanoscale organization; stealth vs. exposure (PEG/corona effects); triggerable/cleavable linkers	Over-functionalization can reduce uptake/escape; receptor saturation; increased off-target uptake; organelle localization must be validated (uptake ≠ organelle delivery)
Peptide decoration	CPP-mediated uptake enhancement; fusogenic/endosomolytic peptides; organelle-targeting peptides (context-dependent)	Peptide amphipathicity; net charge; protease stability; presentation/orientation; stimulus-responsive masking/unmasking	Endosomal entrapment common; proteolysis; immunogenicity; potential membrane toxicity; requires orthogonal localization + function validation

CPPs; Cell-Penetrating Peptides, NLS; nuclear localization signals, TPP; triphenylphosphonium, ROS; reactive oxygen species, NPs; Nanoparticles, pH; potential of hydrogen, ER; endoplasmic reticulum.

### 3.4 Surface architecture and post-cytosolic organelle sorting

The surface architecture of a nanomaterial determines whether an organelle-targeting motif remains accessible and functional after cellular uptake. Relevant variables include ligand identity, density, spacing, orientation, linker length, surface charge, and the extent of steric shielding by polymer coatings or the biomolecular corona. High ligand density may increase multivalent binding, but excessive crowding can reduce ligand accessibility or alter endosomal processing. Similarly, adsorption of serum proteins may mask targeting motifs, whereas cleavable coatings or stimulus-responsive unmasking can expose them after endosomal escape. Organelle targeting therefore depends not only on the presence of a targeting ligand but also on its spatial presentation and availability following cytosolic entry (Albanese et al., 2012; Kern et al., 2021).

After cytosolic release, mitochondrial localization may occur through several mechanistically distinct routes. Lipophilic cations such as triphenylphosphonium do not require recognition by a mitochondrial receptor; instead, they accumulate through electrostatic partitioning driven by the negative mitochondrial membrane potential (Murphy, 2008). By contrast, canonical mitochondrial targeting sequences are generally amphipathic, positively charged peptides that are recognized by receptors of the translocase of the outer mitochondrial membrane, followed by passage through TOM and TIM complexes into mitochondrial subcompartments. This import process depends on peptide accessibility, cargo size, mitochondrial membrane potential, and interactions with cytosolic and mitochondrial chaperones. Importantly, the canonical import machinery is size-constrained, and surface attachment of a targeting peptide does not necessarily permit translocation of an intact nanoparticle; in many systems, the released molecular cargo or a smaller conjugate, rather than the complete carrier, reaches the mitochondrial interior. Mitochondria-penetrating peptides represent a related but distinct approach in which cationic and amphiphilic sequence properties promote interaction with and translocation across mitochondrial membranes without necessarily relying on canonical receptor-mediated import (Horton et al., 2008; Wiedemann and Pfanner, 2017).

Nuclear sorting similarly requires that a nuclear localization signal remain exposed after endosomal escape. Classical NLS motifs are recognized by importin-α, which recruits importin-β and directs the cargo to phenylalanine–glycine-repeat nucleoporins within the nuclear pore complex. The receptor–cargo complex then traverses the nuclear pore, and RanGTP-dependent dissociation releases the cargo in the nucleoplasm. Effective nuclear delivery therefore depends on NLS accessibility and density, cargo hydrodynamic size and deformability, importin engagement, and avoidance of persistent endosomal or perinuclear sequestration. Large intact nanomaterials may be unable to undergo conventional interphase nuclear-pore transport, and apparent nuclear accumulation may instead reflect delivery of released cargo or access during mitotic nuclear-envelope disassembly (Hampoelz et al., 2019; Jans et al., 2019; Nie et al., 2023).

Thus, precise intracellular sorting is produced by a sequence of coordinated events rather than by a single surface ligand. Successful designs must combine cellular uptake, controlled endosomal escape, cytosolic exposure of the targeting motif, compatibility with the relevant organelle-recognition or membrane-partitioning mechanism, and validation that the intact carrier or released cargo reaches the intended subcellular compartment.

### 3.5 Critical comparison of intracellular nanomaterial-targeting strategies

The different strategies used to achieve intracellular and organelle localization provide distinct advantages but also involve important trade-offs. Physicochemical optimization of particle size, charge, shape, and deformability is broadly applicable and relatively simple to implement, but its effects are strongly dependent on cell type, protein-corona formation, and endocytic pathway selection, thereby limiting organelle specificity (Behzadi et al., 2017; Cai et al., 2024). Ligand- and peptide-functionalized systems can improve receptor-mediated uptake or bias localization toward mitochondria or the nucleus; however, their effectiveness depends on ligand density, spatial organization, receptor availability, and successful endosomal escape (Ackun-Farmmer et al., 2020; Kern et al., 2021). Increased cellular uptake therefore does not necessarily translate into greater cytosolic, organellar, or MCS access, because a substantial fraction of internalized nanomaterials may remain trapped within endosomal compartments (Beach et al., 2021).

Stimuli-responsive and charge-reversible platforms provide improved temporal control by activating membrane-disruptive or targeting functions under defined intracellular conditions (Ganta et al., 2008). Nevertheless, heterogeneity in intracellular pH, redox state, and enzyme activity may cause incomplete activation, variable performance, or unintended membrane damage. Multi-organelle and putative MCS-directed systems may coordinate several intracellular pathways, but their greater design complexity also increases manufacturing difficulty, mechanistic ambiguity, and the burden of localization, functional, and safety validation. Table 2 summarizes representative studies showing how particle dimensions, geometry, charge, and surface functionalization influence uptake, trafficking, and organelle localization.

**TABLE 2:** Physicochemical properties and intracellular targeting outcomes of representative nanomaterial formulations.

Nanomaterial	Physicochemical and targeting characteristics	Intracellular target	Main finding	References
Fluorescent polystyrene latex beads	Spherical particles with diameters of 50, 100, 200, 500, and 1,000 nm; no targeting ligand.	Endocytic and endolysosomal pathways	Using B16-F10 murine melanoma cells, Particles ≤200 nm were internalized predominantly through clathrin-mediated endocytosis, whereas 500 nm particles preferentially used a caveolae-associated pathway and showed limited lysosomal delivery.	Rejman et al. (2004)
Citrate-stabilized spherical AuNPs and CTAB-prepared Au nanorods	Spherical AuNPs: 14, 30, 50, 74, and 100 nm; nanorods: 40 × 14 and 74 × 14 nm, with approximate aspect ratios of 3 and 5, respectively. Citrate-stabilized spheres carried a negative surface charge, whereas the rods were CTAB-coated; No organelle-targeting ligand was used.	Cytoplasmic endocytic vesicles; no nuclear entry	Using HeLa cells, uptake was maximal for approximately 50 nm spherical AuNPs. Spherical particles were internalized more efficiently than rods, and lower-aspect-ratio rods showed greater uptake than higher-aspect-ratio rods.	Chithrani et al. (2006)
Tiopronin-coated AuNPs, including fluorescent and oligonucleotide-conjugated derivatives	Approximately spherical negatively charged AuNPs with a size of 2, 6, 10, and 16 nm; Particles were coated with tiopronin and, functionalized with PEG–fluorophore or triplex-forming oligonucleotides.	Nucleus	Using MCF-7 cells and tumor models, the 2 and 6 nm AuNPs showed efficient nuclear entry, whereas 10 and 16 nm particles were largely excluded from the nucleus, demonstrating size-dependent nuclear access.	Huo et al. (2014)
Bare Au nanorods	Rod-shaped particles measuring approximately 36 × 12 nm, with an aspect ratio of approximately 3; ζ-potential: −47.18 ± 4.26 mV; no active targeting ligand.	Macropinosomes and lysosomes	Using SKBR-3 and MCF-7 breast cancer cells, the nanorods were internalized predominantly through macropinocytosis and subsequently detected within macropinosomal and lysosomal compartments.	White et al. (2022)
AgNPs	Approximately spherical particles with a mean TEM diameter of approximately 9 nm; no organelle-targeting ligand reported.	Mitochondrial and ER homeostasis; indirect relevance to ER–mitochondria communication	MCF-7 cells. AgNP exposure induced mitochondrial fragmentation, altered membrane potential, ER stress, and increased spatial association between ER and mitochondrial markers; however, direct MCS targeting was not demonstrated.	Dey et al. (2022)
10% PEI–PLGA nanoparticles	Approximately spherical particles; unloaded formulation: 250.29 ± 1.45 nm; vNAR-loaded formulation: 249.26 ± 2.13 nm; ζ-potential of the unloaded formulation: +6.01 ± 3.86 mV. The cationic surface was provided by PEI, with vNAR incorporated as the delivered protein cargo.	Endosomes followed by enhanced cytosolic cargo delivery	HeLa cells. Relative to anionic PLGA nanoparticles, the cationic formulation enhanced cellular uptake and promoted endolysosomal membrane disruption and intracellular delivery of soluble cargo.	Tracey et al. (2023)
CoQ10-loaded MITO-Porter	Liposomal nanoparticles with a hydrodynamic diameter of approximately 70 nm and a ζ-potential of approximately +15 mV; surface-functionalized with octaarginine peptide for enhanced cellular and mitochondrial delivery; CoQ10 was incorporated as the functional cargo.	Mitochondria	L6 rat skeletal-muscle cells. MITO-Porter-mediated mitochondrial delivery of CoQ10 increased oxygen-consumption rate and enhanced mitochondrial respiratory capacity.	Sato et al. (2024)

AuNPs, gold nanoparticles; CoQ10, coenzyme Q10; CTAB, cetyltrimethylammonium bromide; DLS, dynamic light scattering; ER, endoplasmic reticulum; MCS, membrane contact site; NR, not reported numerically in the primary article; PEI, polyethyleneimine; PLGA, poly (lactic-co-glycolic acid); TEM, transmission electron microscopy; vNAR, variable new antigen receptor.

## 4 Mitochondrial architecture and ER–mitochondria contact-site regulation by nanomaterials

### 4.1 Mitochondria as structural and metabolic regulators of cell fate

The mitochondria are thought to be integrative hubs, connecting cellular bioenergetics with the signaling pathways that control proliferation, differentiation, stress adaptation, and apoptosis. In addition to their production of ATP through oxidative phosphorylation, mitochondrial metabolism produces TCA cycle-derived metabolites (e.g., citrate, α-ketoglutarate, and succinate), which serve as signaling metabolites, modifying chromatin structure and the transcriptional programs of genes (Martínez-Reyes and Chandel, 2020). Mitochondrial respiration interacts with these metabolites through different metabolic-epigenetic interfaces and exerting influence on lineage specification and the decisions to assign identities to cells.

Cellular ATP availability reflects coordinated regulation of substrate flux, electron transport chain activity, and mitochondrial membrane potential. Each of these variables will create energetic and redox (electron) barriers around the cell which will limit the amount of building blocks the cell can make, affecting how cells will transition through cell fate decisions (Zhang et al., 2011). Disruption in mitochondrial energy production will favor either anabolic or catabolic pathways and thus reflect how the cell will either maintain their proliferative potential or transition down the path of differentiation. Thus, the levels of the energy from mitochondria not only represents a permissive factor but also create a key variable that determines whether cells will commit to their fate decisions.

Depending on the concentration, localization and duration, mitochondrial reactive oxygen species (mROS) have context-dependent roles as signaling molecules. Presenting low to moderate levels of mROS regulates redox-dependent signaling pathways and transcription factors that regulate processes that promote differentiation and adaptive responses to stress; whereas when accumulated at elevated or prolonged levels, mROS will activate damage response pathways resulting in senescence or cell death. The duality of mROS as both signaling and toxic molecules provides a means for mitochondria to transduce metabolic and environmental signals into variable cell fate outcomes (Schieber and Chandel, 2014; Zorov et al., 2014).

Changes in metabolic state between cells transitioning from one fate to another typically involve a shift from the use of glycolysis as an energy source to the use of oxidative phosphorylation as an energy source. This metabolic reprogramming is associated with a change in the NAD+/NADH ratio, the flux through the TCA cycle, and the regulation of chromatin-modifying enzymes in a metabolite-dependent manner. This coordinated change allows mitochondria to help actively alter the transcriptional landscape of the cell, supporting the hypothesis that cellular fate transitions are driven by metabolism, rather than being caused by fate transitions (Ito and Ito, 2016).

Importantly, these mitochondrial functions are coordinated partly through ER–mitochondria membrane contact sites, which link mitochondrial state to intracellular signaling and cell-fate regulation. Table 3 summarizes the key strategies and mechanisms through which nanomaterials can regulate mitochondrial functions, providing a framework for understanding how these interventions can modulate cell fate decisions.

**TABLE 3:** Key structural and functional mechanisms of mitochondrial regulation by nanomaterials.

Strategy/mechanism	Key description	Structural/cellular readouts	Challenges/considerations
Mitochondrial targeting	Use of lipophilic cations (e.g., TPP) to exploit mitochondrial membrane potential	Supports verified mitochondrial localization and assessment of redox, cristae, and bioenergetic effects	Endosomal escape, lysosomal avoidance, and validation of true mitochondrial localization
Mitochondria penetrating peptides	Short, cationic peptides that cross both plasma and mitochondrial membranes	Modulate ROS tone, membrane potential, cristae integrity, and mitochondrial function	Peptide design must balance uptake, structural specificity, and membrane compatibility
Mitochondrial-nucleus signaling (mitonuclear communication)	Nanomaterials influence mitonuclear signaling to modulate gene expression	Links mitochondrial structure/function to differentiation, proliferation, survival, and apoptosis	Need for precise structural and transcriptional validation
Nano-regulation of mitochondrial metabolism	Targeting ATP production, redox state, and metabolic flow	Shifts metabolic state and may alter mitochondrial morphology and fate decisions	Balancing bioenergetic remodeling with preservation of organelle integrity
ROS regulation by nanomaterials	Catalytic scavenging/generation of ROS to control cell survival vs. apoptosis	Can mitigate oxidative stress, alter mitochondrial morphology, or induce apoptosis	Context-dependent role of ROS in signaling, morphology, and toxicity
Mitochondrial morphology and dynamics	Nanomaterials can alter mitochondrial morphology to regulate fission/fusion	Alter survival, damage repair, apoptosis, and mitochondrial structural remodeling	Requires structural readouts of fission/fusion, cristae, and mitophagy
Mitochondrial stress and toxicity	Nanomaterial-induced stress can trigger mitophagy and mitochondrial apoptosis	Stress response produces cell death, adaptation, or mitophagy-associated structural remodeling	Stress thresholds require dose-response and ultrastructural validation

TPP; triphenylphosphonium, ROS; reactive oxygen species.

As mitochondrial energy production and redox states influence the cellular transcriptional landscape, mitochondrial dysfunction can initiate retrograde signaling that directly impacts nuclear gene expression. This mitonuclear communication is essential for maintaining cellular homeostasis and controlling fate decisions, which is explored in more detail in Section 5, where we discuss nanomaterials designed to target the nucleus and regulate gene expression.

### 4.2 ER–mitochondria contact sites as nanoscale regulators of mitochondrial function

ER–mitochondria contact sites organize localized calcium and lipid exchange and contribute to mitochondrial division, bioenergetic regulation, redox adaptation, and apoptotic signaling. Their functional output depends not only on contact abundance but also on intermembrane spacing, molecular composition, duration, and subcellular distribution. Representative regulatory assemblies include the VAPB–RMDN3/PTPIP51 complex and PDZD8-associated machinery, while contact-enriched IP_3_ receptors support localized calcium transfer from the ER to mitochondria (Hirabayashi et al., 2017; Katona et al., 2022).

Contact geometry is particularly important. Experimental manipulation of ER–mitochondria spacing showed that an intermembrane distance of approximately 20 nm supports efficient mitochondrial Ca^2+^ uptake and oxidative metabolism, indicating that increased proximity does not necessarily correspond to improved function (Dematteis et al., 2024). ER tubules also define sites of mitochondrial constriction and division, with ER-associated actin remodeling contributing to mitochondrial fission (Friedman et al., 2011; Korobova et al., 2013). These interfaces therefore connect mitochondrial morphology with metabolic regulation and cell-death signaling.

Nanomaterials may influence ER–mitochondria contacts either directly or indirectly. Direct modulation requires evidence that the nanomaterial, its surface components, or its released cargo reaches the interface and alters contact spacing, tether recruitment, contact duration, or contact-dependent molecular exchange. Indirect effects may result from oxidative stress, ER stress, mitochondrial depolarization, altered calcium homeostasis, or membrane remodeling. For example, silver nanoparticles have been reported to disturb both ER and mitochondrial homeostasis, but without direct measurement of contact architecture such findings indicate potential ER–mitochondria dysregulation rather than proven MCS targeting (Dey et al., 2022). Accordingly, claims of direct nano–MCS modulation should combine structural assessment of the contact interface with functional measurements of calcium transfer, lipid exchange, respiration, mitochondrial division, or apoptosis.

### 4.3 Strategies for mitochondrial localization and structural targeting

**FIGURE 3:**
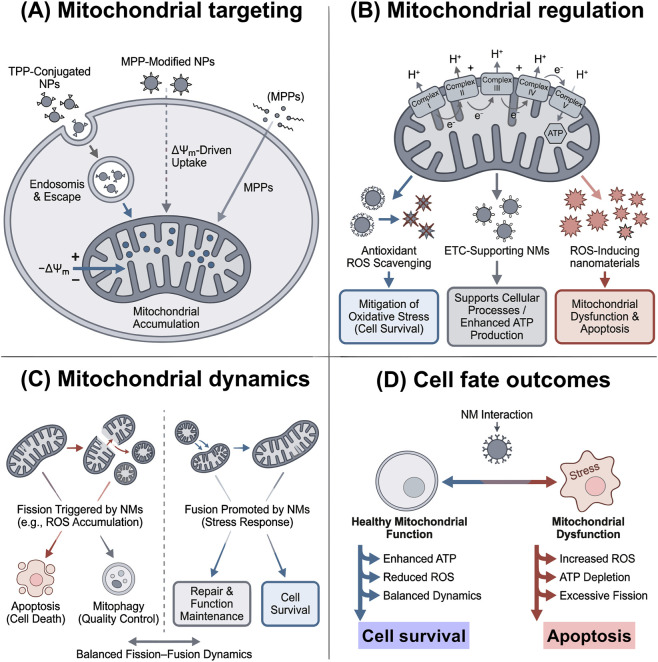
Mitochondrial structure, metabolism, and targeting by nanomaterials. **(A)** Nanomaterials may localize to mitochondria through TPP-conjugated nanoparticles (TPP-NPs), mitochondria-penetrating peptide-modified nanoparticles (MPP-NPs), or other mitochondria-biased surface designs. **(B)** Mitochondrial localization can influence respiration, ATP-linked oxygen consumption, membrane potential, and ROS tone. **(C)** Structural changes in mitochondrial fission/fusion balance and cristae integrity condition survival, adaptation, or apoptosis. **(D)** Mitochondrial dysfunction can transmit structural and metabolic signals to nuclear gene programs.

Mitochondrial targeting strategies exploit the electrochemical properties of mitochondria (notably the negative inner membrane potential), mitochondrial import machinery, and/or organelle-specific membrane interactions (Figure 3A). Delocalized lipophilic cations such as triphenylphosphonium (TPP) exploit the membrane potential to drive several-hundred-fold accumulation within mitochondria, with uptake scaling exponentially with membrane potential rather than ligand–receptor kinetics. Building on this principle, Sulik et al. synthesized TPP-conjugated antibiotics and demonstrated *in vitro* mitochondrial localization and measurable mitochondria-associated cellular effects in multiple cancer cell lines, directly verifying mitochondria accumulation via dye colocalization and cytotoxicity assays (Sulik et al., 2025). In a separate chemical biology study, TPP-functionalized BODIPY dimers exhibited mitochondrial localization and high photodynamic activity in MCF-7 and HeLa cells, supporting the utility of TPP moieties for organelle-targeted bioactive constructs (Kim et al., 2024). Experimental work with TPP-modified protein NPs also confirmed lysosomal escape and specific mitochondrial localization, improving antioxidant activity in epithelial cells (Zhong et al., 2024). These studies support the reproducibility of membrane-potential-driven mitochondrial targeting across different molecular and particulate platforms. Such accumulation, however, should be distinguished from localization at ER–mitochondria interfaces, which requires direct assessment of the contact region and its associated transfer machinery.

Mitochondria penetrating peptides (MPPs) are short synthetic peptides that have amphiphilic and cationic components and can successfully cross plasma membranes and mitochondrial membranes in a sequence dependent manner. Studies performed on synthetic MPPs show that modifying the charge of the peptide and adjusting the balance between hydrophobic/hydrophilic areas of the peptide can be correlated with the efficiency of uptake into mitochondria thereby delivering cytotoxic or bioactive agents to mitochondria after cellular internalization (Zhang et al., 2025). In addition, Mitochondria-targeted tetrapeptides such as SS-31 (Elamipretide) which binds to the cardiolipin lipid found in the inner mitochondrial membrane, have been shown to inhibit ROS production and protect against declines in mitochondrial membrane potential and improve the functional outcomes of oocytes model systems thereby confirming mitochondria target engagement in mammalian cells (Nguyen et al., 2025).

Nanoparticles can also have additional methods to create a bias that allows their accumulation near the mitochondria after cellular uptake by endocytosis, or upon being released directly into the cytosol. Active targeting is achieved by functionalizing NPs surfaces with mitochondrial targeting moieties (e.g., TPP) or peptides to drive organelle-specific interactions post-internalization. Recent work illustrates that TPP-modified polymer/protein nanoparticles exhibit enhanced mitochondrial accumulation compared with unmodified counterparts *in vitro*, resulting in improved mitochondrial ROS mitigation and functional outcomes in cell models (Zhong et al., 2024). Emerging nanosystems that combine cationic motifs with optimized sizes (typically <100–200 nm) show improved post-endosomal escape and mitochondrial association compared with untargeted nanoparticles, confirming the synergistic effect of surface functionalization and particle design on organellar targeting (Huang et al., 2026).

### 4.4 Nano-regulation of mitochondrial metabolism and ultrastructure

Mechanistic regulation of nanomaterials can be done through (i) targeted perturbation of redox/energetic state of mitochondria, (ii) alteration of respiratory chain activity and metabolic flow, and (iii) adaptation of mitochondrial morphology and dynamics. There are two different yet related contexts that have to be differentiated in a mechanistic review: intentional regulation (nano-enabled control of mitochondrial processes), and incidental regulation (mitochondrial perturbation as an exposure or stress response).

The production of mitochondrial ATP can be affected by the modulation of proton-motive force, flux of electron transport or delivery of substrates to the respiratory chain. A clear experimental demonstration is provided by Sato et al. who delivered Coenzyme Q10 directly to mitochondria using the MITO-Porter nanocarrier and quantified bioenergetic effects using extracellular flux analysis; mitochondrial CoQ10 delivery increased oxygen consumption rate (OCR) and enhanced mitochondrial respiratory capacity in L6 skeletal muscle cells (Sato et al., 2024).

At the organelle–pathology interface, Liang et al. developed a mitochondria-targeted fluoropolymer NPs and showed that treatment of foam cells increased ATP concentration, restored membrane potential, and significantly improved basal, maximal, and ATP-linked respiration (ATP production) as quantified by mitochondrial respiration assays, accompanied by ultrastructural rescue of cristae (Liang et al., 2025).

In addition to delivery of antioxidants, recent nano-enabled interventions directly remodel respiratory chain function. Gao et al. designed a mitochondria-targeted black phosphorus/ceria nanozyme and showed that the construct restored mitochondrial complex II activity, reduced oxidative damage, and supported sufficient ATP production during recovery from acetaminophen-induced liver injury, explicitly linking nano-intervention to respiratory complex function rather than nonspecific cytoprotection (Gao X. et al., 2025).

Nanomaterials can shift redox state by catalytic ROS scavenging/generation, altering electron leakage from the respiratory chain, or changing antioxidant buffering capacity, thereby reconfiguring ROS-dependent signaling (survival pathways vs. intrinsic apoptosis/mitophagy). Zhang et al. (2023) reported a mitochondria-targeting Mn_3_O_4_/UIO-TPP nanozyme with sequential SOD-/CAT-like activities; the construct crossed subcellular barriers, eliminated ROS in mitochondria, restored mitochondrial function, and reduced inflammation and apoptosis in chondrocytes, with efficacy demonstrated in an osteoarthritis animal model.

A mechanistic review must also treat mitochondrial effects as a toxicity axis, because many nanomaterials alter respiration without being designed for mitochondrial control. Schilling-Tóth et al. examined gold nanoparticles in hypothalamic POMC cells and found concentration- and time-dependent remodeling of respiration: despite increased maximal respiration in some conditions, ATP-linked OCR (ATP production-associated respiration) decreased, indicating impaired coupling between proton flux and ATP synthase and demonstrating that nanoparticle exposure can induce bioenergetic inefficiency rather than metabolic enhancement (Schilling-Tóth et al., 2025). Figure 3B illustrates how nanomaterials can regulate mitochondrial respiration and ROS production, highlighting their impact on cellular metabolism and ATP production.

Because ER–mitochondria contacts regulate Ca^2+^-dependent metabolism, redox adaptation, and mitochondrial division, parallel measurement of contact architecture and localized Ca^2+^ or lipid transfer can help determine whether observed bioenergetic or ultrastructural effects are MCS-mediated (Katona et al., 2022).

### 4.5 Effects on mitochondrial morphology, survival, and apoptosis

Nano-enabled mitochondrial interventions can bias cells toward survival/adaptation or toward intrinsic cell death, primarily by shifting (a) mitochondrial membrane potential and permeability transition propensity, (b) local mitochondrial redox tone (mtROS vs. antioxidant buffering), and (c) the probability and kinetics of mitochondrial outer membrane permeabilization (MOMP) with downstream cytochrome-c–Apaf-1 apoptosome assembly and caspase-9/3 activation. Contemporary cell-death syntheses emphasize that mitochondrial dynamics and ultrastructure (cristae remodeling, fission/fusion state) are not passive correlates but mechanistically condition MOMP competence and the efficiency of intermembrane-space protein release, thereby sharpening or blunting apoptotic commitment thresholds (Khatun et al., 2024).

When nanomaterials are designed to reduce mtROS and stabilize respiratory function, survival outcomes commonly align with preserved membrane potential, maintained electron-transport activity, and suppression of MOMP initiation. For example, Zhao et al. (2026) engineered a TPP-modified, mitochondria-targeted NPs for dry-eye disease and showed strong ROS-scavenging capacity with protection of mitochondrial function *in vitro* and *in vivo*, consistent with a survival-shift through mitochondrial stabilization rather than cytotoxic stress.

Conversely, when mitochondrial stress exceeds buffering capacity, through persistent mtROS generation, calcium overload, respiratory inhibition, or direct mitochondrial delivery of death-amplifying or structure-perturbing cargos, nano-exposures and some bioactive nano-platforms can push cells toward intrinsic apoptosis or related mitochondria-linked regulated death modalities. In a mechanistic toxicology study, Sun et al. exposed TM4 cells to TiO_2_ NPs and demonstrated that ROS accumulation was coupled to reduced MCUb expression, mitochondrial Ca^2+^ overload, loss of mitochondrial membrane potential, and increased apoptotic signaling proteins (including Bax/Cytochrome-c/Caspase-9/3 axis), supporting a ROS/Ca^2+^/mitochondrial apoptosis cascade (Sun et al., 2025). In oncology-directed designs, Jiang et al. developed a nanocomposite (Lipo-Ele@CuO_2_) that delivers copper to mitochondria and induces mitochondria-mediated cuproptosis with radiation-associated stress responses, illustrating how mitochondria-targeted metal stress can be harnessed therapeutically to drive lethal mitochondrial signaling rather than protect it (Jiang et al., 2025). These findings justify treating “energetic reserve” and “redox capacity” as state-dependent modifiers that determine whether nano-perturbations remain within adaptive signaling ranges or cross a threshold into intrinsic apoptosis or other mitochondria-driven regulated death programs.

ER–mitochondria contacts can modify survival–death thresholds by controlling localized Ca^2+^ transfer, mitochondrial constriction and division, bioenergetic adaptation, and apoptotic competence. Excessive or prolonged coupling may promote mitochondrial Ca^2+^ overload, whereas insufficient coupling may impair Ca^2+^-supported metabolism and stress adaptation (Carpio et al., 2021). Figure 3C illustrates the relationship between mitochondrial fission–fusion dynamics and cell survival or apoptosis, and Figure 3D summarizes cell-fate consequences of mitochondrial dysfunction.

## 5 Nuclear-targeted nanomaterials and nuclear responses downstream of organelle contact-site signaling

**FIGURE 4:**
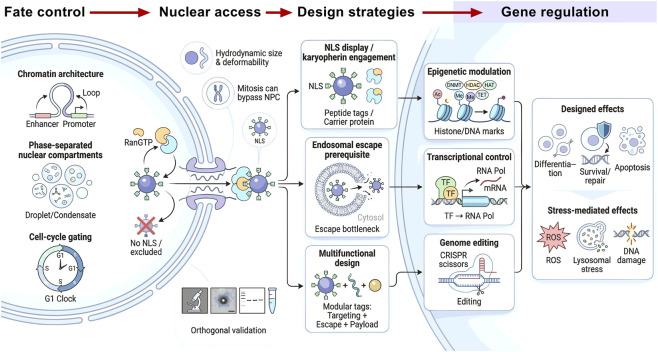
Nuclear-targeted nanomaterials regulate chromatin organization, gene expression, and cell identity through nuclear access, payload engagement, and context-dependent structural outcomes. Nuclear organization provides a control layer for cell fate through nuclear-pore transport, chromatin topology, epigenetic state, and transcriptional regulation. Apparent nuclear localization should be distinguished from perinuclear accumulation or mitotic nuclear-envelope breakdown and should be confirmed with orthogonal structural methods.

The nucleus is a central integrator of cell fate because it converts transient signaling inputs into stable transcriptional and epigenetic programs. For nuclear-targeted nanomaterials, achieving meaningful control requires more than delivering a payload into the nucleoplasm; it requires navigating the endosomal escape bottleneck, engaging the importin/karyopherin system, and overcoming the selectivity of the NPC. Importantly, apparent “nuclear localization” can reflect perinuclear accumulation or cell-cycle–dependent nuclear envelope breakdown, so claims of interphase nuclear import benefit from orthogonal validation. Once nuclear access is established, nanomaterials can modulate gene regulation through epigenetic remodeling, transcriptional control, or genome editing, with outcomes that range from differentiation to survival/repair or apoptosis. Figure 4 summarizes this pathway from nuclear access constraints to downstream gene-regulatory mechanisms and highlights the distinction between designed effects and stress-mediated responses.

Nuclear responses may arise through direct intranuclear delivery or indirectly through signals generated at organelle contact sites. ER–mitochondria contacts can influence nuclear transcription through Ca^2+^-dependent signaling, redox changes, metabolic intermediates, and mitochondrial stress responses, whereas lysosome-associated signaling can regulate transcription factors such as TFEB. Accordingly, changes in nuclear gene expression following nanomaterial exposure should not automatically be attributed to direct nuclear targeting, because they may instead reflect MCS-dependent or organelle-stress-mediated retrograde signaling.

### 5.1 The nucleus as a structural control center of cell fate

Nuclear organization and gene regulation ultimately stabilize cell fate decisions. Nuclear processes integrate transcription factor activity, chromatin topology, and epigenetic modifications into durable gene expression programs, which define lineage commitment and functional identity (Misteli, 2020). In this regard, higher-order chromatin structures; such as three-dimensional genome folding, enhancer-promoter interactions, and phase-separated nuclear compartments; have emerged as a mechanistic layer that limits which regulatory interactions can physically occur and therefore what transcriptional states can be achieved during developmental transitions of fate (Pelham-Webb et al., 2021). It has been shown that there is a dynamic change in three-dimensional chromatin organization during embryonic development, differentiation, or reprogramming (Bonev et al., 2017). Furthermore, phase separation is now becoming an increasingly important physical driver of chromatin folding and gene regulatory control (Gibson et al., 2019).

Beyond transcription and chromatin structure, cell-cycle governance is tightly coupled to fate control (Soufi and Dalton, 2016). Remodeling of G1 duration and cyclin/CDK logic is repeatedly observed during exit from pluripotency and early differentiation, and mechanistic cross-links between pluripotency networks and cell-cycle regulation provide a route by which nuclear state transitions become temporally gated (Ter Huurne et al., 2017).

Therefore, the main challenge for intracellular nanotechnology will be to selectively gain access to the nucleus while either maintaining or altering (deliberately) the chromatin and transcriptional landscape and not just delivering a functional payload into the nucleoplasm (Nie et al., 2023).

Nuclear organization is also responsive to extranuclear signals. Contact-site-regulated Ca^2+^ transfer, mitochondrial redox state, and metabolite availability can influence transcription factors, chromatin-modifying enzymes, and stress-responsive gene programs (Quirós et al., 2016; Martínez-Reyes and Chandel, 2020). Nano-induced chromatin or transcriptional changes may therefore arise downstream of altered organelle communication rather than from direct interaction with nuclear structures.

### 5.2 Nuclear transport mechanisms and nuclear-pore constraints

Access to the nucleus is controlled by the nuclear envelope and NPC, which act as a selective barrier as opposed to a passive opening. Intrinsically disordered FG-repeat nucleoporins largely control the permeability of NPCs, providing for rapid transport of receptor-cargo complexes while also preventing most large macromolecules that do not have the appropriate transport receptors from entering the nucleus (Hampoelz et al., 2019).

The process of selective transport is further refined through the recognition of nuclear localization signals (NLS) and export signals by karyopherins (importins and exportins) (Chook and Süel, 2011). Karyopherins bind to and engage in multiple interactions with the FG-repeats within the NPC, thus defining the specificity and efficiency of the nuclear import and export processes (Tu and Musser, 2011).

For cargo targeting the nucleus, NLS motifs are crucial for recognition by the importin system, providing a widely utilized entry route. The coordination of NLS recognition, importin-α/β interaction, and RanGTP gradients ensures the directional transport and precise release of cargo within the nucleus. These mechanistic principles are directly relevant to the design of nanomaterials intended for nuclear delivery (Poon and Jans, 2005; Jans et al., 2019).

There are two primary engineering challenges related to the constraints of nuclear transport that must be addressed when designing organelle-targeted nanomaterials. First, they must navigate to the perinuclear space without getting sequestered in endolysosomal compartments (as described in Section 3). Second, the engineering of the interfaces used to transport the cargo (e.g., NLS displays, receptor recruitment strategies, or modulation of transitory barriers) must ensure structural integrity of the nuclear pore complex when allowing cargo to pass through the NPC (Schuller et al., 2021; Zimmerli et al., 2021).

Import into the nucleus is highly size- and mechanism-dependent: passive diffusion through the NPC is effectively restricted to small proteins, whereas larger cargos require active transport mediated by NLS recognition and importin/karyopherin engagement. For nanomaterials, the relevant ‘size’ is the effective hydrodynamic diameter and deformability (including corona and coating), not simply the inorganic core dimension. Importantly, many reports of nuclear nanoparticle accumulation may reflect cell-cycle–dependent access during mitosis, when the nuclear envelope transiently disassembles, rather than interphase NPC-mediated import. Accordingly, claims of intranuclear localization should be supported by orthogonal validation beyond conventional fluorescence microscopy, as perinuclear or nuclear-envelope–associated signal is frequently misclassified as nucleoplasmic entry. Robust confirmation can include super-resolution imaging, TEM/EM, and biochemical nuclear fractionation with stringent contamination controls.

### 5.3 Nanomaterial strategies for nuclear localization and structural engagement

Effective nuclear targeting of nanomaterials is central to regulating gene expression and altering cell identity, because achieving defined structural or reprogramming effects requires delivery of functional payloads directly into the nuclear compartment and engagement with DNA or chromatin machinery (Salatin and Jelvehgari, 2017). Contemporary experimental strategies combine engineered nanoparticle design with mechanisms of nuclear import to surmount cytoplasmic barriers and exploit endogenous transport pathways for intranuclear access (Allen and Yokota, 2024).

A robust strategy for nuclear entry leverages molecular motifs recognized by the nuclear import machinery. Experimental studies have shown that nanocarriers modified with ligands such as NLS or peptides that mimic import receptor binding can engage karyopherin-mediated transport and enhance nuclear accumulation of functional cargoes in live cells. For example, Delille et al. synthesized iron oxide NPs coated with sulfobetaine-phosphonate block copolymers and demonstrated efficient targeting of these probes into the nuclei of living cells, enabling magnetic micromanipulation of a specific genomic locus under an external field (Delille et al., 2023). Another recent study employed phenylboronic acid-modified linear polyethylenimine (LPBA) to achieve targeted nuclear delivery of gene editing and microRNA imaging agents (Wu et al., 2025). LPBA-based nanomaterials facilitated efficient intranuclear localization of functional cargos and enabled real-time imaging of microRNA within the nucleus, illustrating the feasibility of designing polymer-based nanocarriers with inherent nuclear specificity for modulation of gene regulatory networks.

Active nuclear targeting can also be accomplished by exploiting endogenous transport pathways that normally mediate protein import. While classical studies used cell-penetrating peptides to enhance nuclear uptake, contemporary designs focus on integrating similar sequences into nanostructures to improve importin engagement without compromising carrier integrity. Although earlier work demonstrated proof-of-principle for gold NPs conjugated with TAT peptides to enter the nucleus of human fibroblasts, the current trend is toward combining peptide motifs with multifunctional nanocarrier platforms to achieve higher transfection efficiency and gene modulation outcomes. These experimental strategies integrate features that facilitate endosomal escape, cytoplasmic trafficking, and engagement with the NPC for import (De la Fuente and Berry, 2005).

Nanocarriers that deliver genetic material to the nucleus have been developed to modulate gene expression in cell and tissue models. For example, Li et al., reported very high transfection performance in a colon cancer model, quantified primarily by functional reporter expression (i.e., the percentage of cells exhibiting transgene expression) at defined post-delivery time points. Because such functional readouts do not necessarily establish that intact carriers traversed the NPC during interphase, it is important to distinguish transfection outcomes from direct evidence of intranuclear carrier localization (e.g., nuclear fractionation with contamination controls or high-resolution imaging). In proliferating systems, apparent ‘nuclear access’ may also be facilitated by cell-cycle–dependent nuclear envelope dynamics during mitosis rather than *bona fide* interphase NPC transit. Nevertheless, the design principles highlighted in these studies, integrating endosomal escape, cytosolic trafficking, and nuclear import motifs, continue to inform current platforms aimed at improving the efficiency and specificity of nuclear delivery (Li et al., 2017).

### 5.4 Nano-induced regulation of chromatin structure and gene expression

Nanomaterials can influence gene expression through both direct and indirect routes. Direct mechanisms include nuclear delivery of transcriptional or epigenetic effectors and targeted modulation of chromatin-associated machinery (Brzóska et al., 2019; Pogribna et al., 2020). By contrast, many observed transcriptional and epigenetic changes are indirect, stress-mediated effects arising from metabolic disruption, ROS generation, lysosomal perturbation, or activation of DNA-damage and inflammatory signaling. While these stress-mediated responses can reshape chromatin accessibility and gene programs, they are typically less specific and can impose narrow dose windows and safety trade-offs, limiting their suitability as deliberate “cell fate control” strategies (Sierra et al., 2016).

For example, mitochondrial stress caused by nanomaterials has been shown to trigger a cascade of events leading to epigenetic changes in the nucleus, such as altered histone acetylation and DNA methylation patterns, highlighting the role of nanomaterial-induced cellular stress in regulating gene expression (Brzóska et al., 2023). These findings emphasize the need to consider the full spectrum of organelle interactions when assessing the impact of nanomaterials on gene regulation (Manke et al., 2013).

Nanomaterials designed for nuclear targeting can also directly influence transcriptional control by delivering transcription factors or co-regulators into the nucleus, thereby modulating gene expression (Lee et al., 2025). Additionally, nanomaterials can alter NPC function, which regulates the import and export of transcription factors, further modulating gene expression (Zimmerli et al., 2021).

Moreover, nanomaterials can affect chromatin accessibility by inducing epigenetic modifications that influence transcription factor binding, providing a mechanism for altering gene expression at specific genomic loci. For example, gold NPs conjugated with small RNA molecules can alter chromatin condensation, leading to changes in gene expression in human cancer cells.

Nanomaterials also influence cell-cycle dynamics and cell fate decisions by modulating the G1 phase duration and regulating cell-cycle checkpoints, particularly during the transition from pluripotency to differentiation (Soufi and Dalton, 2016). For instance, histone deacetylase inhibitors delivered by nanoparticles have been shown to promote differentiation of human induced pluripotent stem cells (iPSCs) by activating lineage-specific transcription programs.

In summary, nuclear-targeted systems can regulate gene expression only when they combine efficient endosomal escape, verified nuclear entry, appropriate engagement with importin/NLS pathways, and controlled interaction with chromatin or transcriptional machinery. Table 4 summarizes direct nuclear-targeting strategies together with indirect organelle-to-nucleus signaling mechanisms.

**TABLE 4:** Key mechanisms of direct nuclear targeting and indirect organelle-to-nucleus signaling by nanomaterials.

Strategy/mechanism	Key description	Structural and gene-regulatory readouts	Challenges/considerations
Nuclear transport mechanisms	Nanomaterials utilize NLS motifs and karyopherins to gain access to the nucleus	Verified nuclear entry enables analysis of chromatin organization and gene expression	Selective transport, avoiding endolysosomal sequestration, and distinguishing intranuclear from perinuclear signal
Nanomaterial design for nuclear targeting	Nanocarriers modified with NLS or peptides mimic nuclear import machinery	Supports nuclear localization, gene regulation, and analysis of differentiation or proliferation	Requires efficient endosomal escape, carrier integrity, and structural nuclear validation
Chromatin modifications	Nanomaterials can alter histone modification and DNA methylation	Impacts chromatin state, transcriptional regulation, and cell identity	Epigenetic and structural effects may vary with particle size and functionalization
Epigenetic regulation	Nanomaterials may trigger metabolic or structural stress leading to chromatin remodeling	Induces epigenetic changes that affect gene expression at specific loci	Variability in stress and morphology responses across cell types
Nuclear-cytoplasmic transport	Nanomaterials engage importins/exportins for nuclear localization and cargo delivery	Modulates gene expression by altering transcription-factor transport and nuclear organization	Nuclear import varies with effective size, deformability, and cell-cycle state
MCS-dependent organelle-to-nucleus signaling	Nanomaterials alter Ca^2+^ transfer, redox balance, metabolic intermediates, ER-stress pathways, or lysosome-dependent transcriptional signaling without requiring direct nuclear entry	Changes in transcription-factor activity, chromatin state, stress-response programs, and cell-fate-associated gene expression	Nuclear responses alone do not prove MCS involvement; parallel structural or functional evidence of contact-site modulation is required
Gene editing with nanomaterials	Nanomaterials deliver gene-editing tools such as CRISPR/Cas9 to the nucleus	Enables genome modulation that requires nuclear and chromatin structural validation	Precision of nuclear targeting, editing specificity, and genome integrity are key challenges

## 6 Nanomaterial interactions with membrane contact-site networks and multi-organelle crosstalk

### 6.1 Organelle crosstalk in cellular fate and tissue organization

**FIGURE 5:**
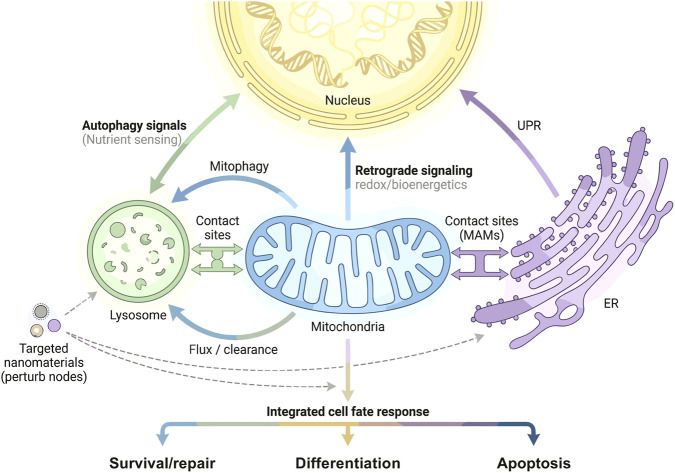
Organelle crosstalk integrates mitochondrial, lysosomal, ER, and nuclear pathways to shape cell fate and tissue organization. Mitochondria link bioenergetic and redox state to nuclear transcriptional programs, lysosomes regulate autophagy and mitophagy, and ER-organelle contact sites coordinate calcium and lipid signaling. Nanomaterials can perturb or probe this network, but structural interpretation requires separating intended organelle engagement from nonspecific stress.

The mitochondria, nucleus, lysosomes, and endoplasmic reticulum (ER) communicate through direct contact sites and retrograde signaling pathways to coordinate cell fate decisions (Figure 5). For example, the mitochondria-nucleus signaling axis is critical in linking mitochondrial bioenergetics, redox state, and stress adaptation to nuclear gene expression (Trevisan et al., 2018). Disruption in one organelle often triggers compensatory mechanisms in others, a phenomenon known as organelle crosstalk. Recent advances have shown that nanomaterials, when engineered to target multiple organelles, can perturb this network in a way that shifts the entire cellular response, enhancing structural and functional outcomes (Li et al., 2024; Singh, 2024). Within this broader communication network, MCSs provide direct nanoscale interfaces for ion transfer, lipid exchange, organelle positioning, membrane remodeling, and stress signaling. Nanomaterials may influence these interfaces either by engaging contact-site machinery directly or indirectly through altered organelle function, trafficking, membrane integrity, or cellular stress. Therefore, evidence of multi-organelle effects should be distinguished from direct MCS modulation unless a defined contact interface and its associated structural or functional changes are demonstrated.

For instance, mitochondrial dysfunction can induce retrograde signaling that activates nuclear transcriptional programs responsible for apoptosis or cell survival. Nanomaterials that target both mitochondria and nucleus can modulate this mitonuclear communication, influencing cell fate in a coordinated manner (Allemailem et al., 2021). Recent studies have demonstrated that mitochondria-targeted nanomaterials can reduce mitochondrial oxidative stress, thereby altering gene expression related to cell survival and proliferation in cancer cells (Yang et al., 2025). Additionally, lysosomal disruption caused by lysosome-targeted nanomaterials can amplify mitochondrial stress, triggering mitophagy and influencing nuclear gene activity to drive cell death or differentiation in cell and tissue models (Chen et al., 2022).

### 6.2 Mitochondria-nucleus signaling axis: nanomaterials as structural modulators

**FIGURE 6:**
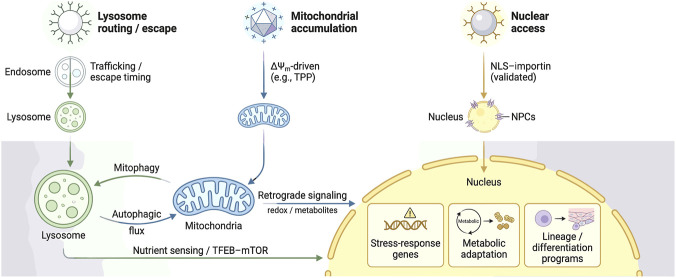
Multi-organelle targeting rewires lysosome-mitochondria-nucleus crosstalk to reshape cellular structure and nuclear gene programs. Nanomaterial designs can engage intracellular intervention points including endosomal trafficking, lysosome avoidance or release, mitochondrial localization, and nuclear delivery. Coordinated effects should be assessed through structural, functional, and longitudinal assays that connect subcellular localization to cell and tissue outcomes.

The ability of mitochondria and nucleus to communicate and influence cell fate is one of the most powerful aspects of multi-organelle targeting by nanomaterials. Mitochondria modulate nuclear gene expression through retrograde signaling, which is tightly linked to the cell’s metabolic state and energy production (Da Cunha et al., 2015). Recent research has revealed that nanomaterials targeting mitochondria can influence nuclear transcription, not only by directly affecting mitochondrial bioenergetics but also by modulating ROS production and redox-sensitive pathways (Shadel and Horvath, 2015). Figure 6 highlights how mitochondrial bioenergetics/redox changes can be transmitted to nuclear gene programs following nanomaterial intervention.

For example, lipophilic cation-based nanomaterials like TPP-conjugated NPs have been shown to accumulate in mitochondria, reduce ROS levels, and induce nuclear gene expression that promotes cell survival or differentiation (Zielonka et al., 2017). This interaction highlights the critical synergy between mitochondrial function and nuclear gene regulation in determining cell fate. The combination of mitochondrial and nuclear targeting enables nanomaterials to modulate both bioenergetic states and gene expression, making them potent tools for cancer cell biology and regenerative bioengineering.

### 6.3 Lysosome-mitochondria-nucleus interaction: a network of cellular quality control

The lysosomes play a pivotal role in cellular homeostasis and stress adaptation by regulating autophagy, mitophagy, and lysosomal biogenesis. Recent studies have highlighted how lysosome-mitochondria crosstalk can influence nuclear gene expression via autophagic flux and mitophagy (Chen et al., 2022). Lysosomal targeting by nanomaterials can alter mitochondrial dynamics, leading to mitophagy and metabolic reprogramming, which in turn modulate nuclear gene expression related to cell survival and differentiation.

For instance, lysosome-targeted NPs have been shown to induce mitophagy, which results in the removal of damaged mitochondria and restoration of mitochondrial function, leading to enhanced gene expression and cellular repair (Zhang et al., 2024). Conversely, lysosomal impairment caused by nanomaterial exposure can compromise mitochondrial maintenance and disrupt nuclear transcriptional programs, leading to cell death (Stern et al., 2012). This lysosome-mitochondria-nucleus interaction underscores the importance of multi-organelle targeting in achieving synergistic structural and functional outcomes.

Mitochondria–lysosome communication also occurs through regulated contacts that coordinate mitochondrial division, ion handling, and quality control. Rab7 activity contributes to contact formation, whereas TBC1D15-dependent Rab7 hydrolysis promotes contact untethering and mitochondrial fission (Wong et al., 2018). Nanomaterial studies seeking to implicate this pathway should assess contact abundance or duration together with the relevant fission, ion-transfer, or quality-control response.

### 6.4 Coordinated targeting of mitochondria, nucleus, and lysosomes

Preclinical studies suggest that multifunctional nanomaterials capable of influencing more than one organelle may produce coordinated cellular effects. However, most available evidence is derived from cellular or early experimental models, and simultaneous localization to multiple organelles, direct modulation of their communication, and causal involvement of specific contact sites are rarely demonstrated.

The simultaneous targeting of mitochondria, nucleus, and lysosomes offers a synergistic approach to regulating cellular processes. Nanomaterials designed to interact with multiple organelles can exploit pathway coupling, where modulating one organelle’s function triggers coordinated changes in the activity of other organelles. For example, mitochondria-targeted nanomaterials that alter mitochondrial dynamics can induce mitophagy in lysosomes while simultaneously modulating nuclear gene expression to enhance apoptotic or repair-associated cellular outcomes (Marrache and Dhar, 2012).

This integrated approach ensures that nanomaterials do not just target a single organelle but instead reshape cellular networks, offering a more comprehensive strategy for cell- and tissue-level investigation. Recent experimental studies have shown that multi-functional nanomaterials, capable of targeting both mitochondria and nucleus, can lead to synergistic effects, such as enhanced apoptosis in cellular models or promoted stem cell differentiation in regenerative bioengineering (Gou et al., 2020; Zong et al., 2025). These findings remain primarily preclinical and should not be interpreted as evidence of direct MCS targeting unless the relevant contact interface is measured.

### 6.5 Multi-organelle targeting in stem-cell and tissue-regenerative contexts

Applications of multi-organelle targeting in stem-cell and regenerative contexts remain exploratory. Available studies suggest that coordinated modulation of mitochondrial metabolism, lysosomal quality control, and nuclear gene regulation may influence differentiation and tissue repair; however, durable regenerative efficacy, long-term safety, and direct involvement of defined MCSs require further validation in physiologically relevant models.

In regenerative bioengineering, coordinated organelle modulation may support stem-cell survival, metabolic adaptation, and lineage commitment. Mitochondria-targeted nanoparticles have been investigated to improve mitochondrial function, whereas nuclear-directed systems may deliver regulators of differentiation and repair programs (Singh et al., 2024).

Lysosome-directed materials may also influence autophagic flux and stress resilience, processes relevant to tissue repair and wound healing (Bi et al., 2025). The therapeutic value of these approaches will depend on demonstrating controlled pathway modulation rather than lysosomal injury, together with durable tissue-level benefit.

In conclusion, communication between mitochondria, the nucleus, and lysosomes is central to the organization of cell fate decisions and tissue-relevant cellular behavior. Nanomaterials that are engineered to engage multiple organelles can be interpreted as structural perturbation tools that reveal how organelle morphology, intracellular trafficking, mitophagy, chromatin state, and stress adaptation are coupled. Table 5 summarizes the key strategies and mechanisms by which nanomaterials modulate organelle interactions, with emphasis on structural and functional readouts rather than nonspecific treatment claims.

**TABLE 5:** Key structural mechanisms of membrane contact-site regulation and multi-organelle crosstalk by nanomaterials.

Strategy/mechanism	Key description	Structural/cellular readouts	Challenges/considerations
Mitochondria-nucleus signaling	Nanomaterials modulate mitonuclear communication to regulate gene expression	Coordinates cell fate, morphology, proliferation, differentiation, survival, and apoptosis	Simultaneous localization and structural validation are complex
Mitochondria and ROS regulation	Mitochondria-targeted nanomaterials reduce ROS levels and influence gene expression	Alters bioenergetic state, morphology, survival, and differentiation in stem-cell models	Need for precise ROS modulation with structural safety readouts
Lysosome-mitochondria crosstalk	Lysosomal targeting modulates mitophagy and mitochondrial dynamics	Regulates survival, stress adaptation, mitophagy-associated morphology, and gene expression	Disruption in lysosomal function can impair mitochondrial integrity and tissue-relevant cell behavior
Defined MCS modulation	Nanomaterials directly or indirectly alter ER–mitochondria, mitochondria–lysosome, ER–endosome, or ER–lysosome interfaces	Changes in contact abundance, length, spacing, duration, tether localization, Ca^2+^ or lipid transfer, mitochondrial division, lysosomal repair, and contact-dependent stress signaling	Organelle localization or downstream functional effects alone do not establish MCS engagement; combined structural, functional, and preferably causal validation is required
Multi-organelle targeting synergy	Simultaneous targeting of mitochondria, nucleus, and lysosomes	Coordinated outcomes: apoptosis, differentiation, stress adaptation, or repair-associated morphology	Managing simultaneous organelle modulation and structural specificity is challenging
Multi-organelle targeting in regenerative bioengineering	Nanomaterials are evaluated for effects on stem-cell differentiation and tissue repair architecture	Links mitochondrial morphology/function, gene expression, and lysosomal activity in stem-cell models	Need for optimized designs and histological readouts for regenerative outcomes

Accordingly, the mechanistic interpretation of multi-organelle nanomaterial effects should follow a hierarchical framework: organelle localization, alteration of a defined contact-site structure, modulation of contact-dependent molecular exchange or signaling, and demonstration that the resulting cellular outcome depends on the relevant MCS machinery. This distinction separates general multi-organelle perturbation from genuine MCS-directed regulation.

## 7 Challenges in achieving morphological and MCS-specific precision in organelle targeting

The central challenge is not merely to deliver nanomaterials to intracellular compartments, but to demonstrate their localization and effects with convincing structural, morphological, and functional evidence. Precision requires verified organelle localization, controlled alteration or preservation of organelle architecture, separation of intentional regulation from nonspecific toxicity, and—in MCS-directed systems—evidence that a defined contact interface or contact-dependent process has been engaged.

### 7.1 Organelle specificity, off-target localization, and structural artifacts

One of the biggest challenges in organelle-targeted nanomaterials is achieving high-level specificity for a given organelle such as mitochondria or the nucleus. Nanomaterials often possess surface characteristics and structural features that produce nonspecific interactions across multiple organelles, leading to off-target localization, altered organelle morphology, or toxicity. This is especially important for mitochondria, which couple bioenergetics to cristae organization and apoptosis (Scorrano et al., 2002), and for nuclei, where nuclear-pore transport, chromatin structure, and genome stability must be preserved or deliberately modulated (Ibarra and Hetzer, 2015).

There are still considerable challenges in demonstrating that a particular nanomaterial truly localizes to one organelle without accumulating in unintended compartments such as lysosomes, endoplasmic reticulum, or perinuclear vesicles. For this reason, claims of organelle targeting should distinguish uptake from localization and localization from structural engagement (Malatesta, 2021). Electron microscopy, super-resolution imaging, biochemical fractionation with contamination controls, and quantitative colocalization can help separate true organelle association from imaging artifacts or stress-induced redistribution (Dunn et al., 2011; Malatesta, 2021).

These limitations are even more important when evaluating MCS engagement. Apparent overlap between two organelle markers and a nanomaterial signal does not establish localization within the narrow intermembrane space of a contact site. Reliable assessment should combine high-resolution or ultrastructural imaging with quantitative measurements of contact abundance, length, spacing, and subcellular distribution. Where possible, contact-site reporters, electron microscopy, or correlative imaging should be complemented by functional measurements of the relevant ion, lipid, or signaling exchange.

Targeting ligands such as NLS motifs and TPP can improve nuclear and mitochondrial localization, respectively. NLS motifs act through importin/karyopherin recognition and NPC transport (Lange et al., 2007), whereas TPP accumulates primarily through membrane-potential-dependent partitioning (Murphy, 2008). Hydrodynamic size, surface charge, ligand presentation, endosomal escape, and cytosolic availability determine whether these cues remain functional or whether the carrier is instead routed to lysosomes. Multifunctional systems may add staged or conditionally exposed motifs, but the resulting increase in complexity also raises the risk of off-target uptake and stress-mediated effects.

### 7.2 Multi-organelle targeting and interference with native cell architecture

Although multi-organelle targeting may reveal coordinated structural regulation, it also adds a high level of complexity. The ability of nanomaterials to interact with mitochondria, nuclei, and lysosomes without disrupting their native architecture is a significant challenge (Quirós et al., 2016; Wong et al., 2018). For example, mitochondrial perturbation can reshape nuclear transcriptional programs, while lysosomal stress can secondarily alter mitochondrial morphology, mitophagy, and nuclear gene activity (Wong et al., 2018).

For MCS-directed systems, preserving native architecture also requires maintaining the physiological balance between contact formation and dissolution. Excessive stabilization of an interface may promote Ca^2+^ overload, abnormal lipid redistribution, impaired organelle motility, or defective autophagic flux, whereas excessive contact disruption may reduce molecular exchange and organelle coordination. Therefore, successful MCS modulation should produce a controlled and reversible change in contact-site behavior rather than persistent tethering, widespread membrane deformation, or generalized organelle injury.

The use of sequential targeting strategies provides a possible solution to this challenge. Using this method, nanomaterials are engineered to target multiple organelles in a specific sequence. A more realistic interpretation of sequential multi-organelle targeting is not to “activate” or “degrade” lysosomes as a generic enabling step, but to engineer controlled intracellular trafficking and staged payload release. In practice, this can be achieved by timed endosomal escape, lysosome-avoidance routing, or triggered release mechanisms that minimize lysosomal sequestration before subsequent targeting to mitochondria or the nucleus (Selbo et al., 2010). Such sequencing can reduce cross-organelle interference by ensuring that each payload reaches the appropriate compartment under defined conditions and time windows. Importantly, overt lysosomal damage is not a neutral intervention: it can activate inflammasome signaling, disrupt autophagy/mitophagy flux, and induce secondary mitochondrial and nuclear stress responses, which undermines precision and confounds interpretation of “organelle-specific” effects (Hornung et al., 2008).

### 7.3 Toxicity, biocompatibility, and preservation of cell and tissue structure

A key interpretive challenge is that many pathways that alter gene expression and organelle morphology, such as ROS production, lysosomal destabilization, mitochondrial dysfunction, and DNA-damage signaling, are also canonical toxicity mechanisms (Nel et al., 2006). Therefore, stress-mediated transcriptional or structural remodeling should not be presented as controlled cell-fate regulation unless it occurs within a defined dose window, with acceptable viability and preserved long-term cellular architecture, genomic stability, epigenomic stability, and tissue compatibility (Soenen et al., 2011).

Nanomaterials can have toxic effects on living systems even when they are designed to target endogenous organelles such as mitochondria or nuclei. Mitochondrial-targeting systems may induce oxidative stress, cristae disruption, or altered membrane potential; nuclear-targeting systems may disturb DNA, chromatin organization, nuclear-pore function, or genome stability. Therefore, designs intended for biological applications should include morphological, histological, and ultrastructural safety readouts in addition to conventional viability assays.

For putative MCS-directed nanomaterials, safety assessment should also determine whether contact-site remodeling remains within a physiological and reversible range. Persistent changes in contact abundance, spacing, or tethering may disturb Ca^2+^ homeostasis, lipid distribution, mitochondrial metabolism, lysosomal repair, or autophagic flux even when short-term cell viability appears preserved. Dose–response and recovery studies should therefore combine conventional toxicity endpoints with longitudinal assessment of contact architecture and contact-dependent function.

If nanomaterials are not designed and validated for biocompatibility, they may cause cytotoxicity, mutagenesis, inflammation, or persistent distortion of cellular and tissue architecture. Demonstrating safety therefore requires more than short-term uptake and viability testing; it should include dose-response analysis, recovery studies, and longitudinal assessment of organelle morphology and tissue structure.

One option for reducing toxicity is to design biodegradable nanomaterials that degrade after completing their intended subcellular function. Another strategy is to modify nanomaterial surfaces, such as through PEGylation or stimulus-responsive shielding, to reduce off-target interactions and improve compatibility with cellular and tissue architecture.

### 7.4 Scalability, manufacturing, and reproducibility of structural outcomes

The progress made so far in nanomaterial design has been significant; however, scaling precision-targeted systems while maintaining reproducible subcellular localization and structural outcomes remains a major challenge. Large-scale production must ensure that each batch maintains consistent size distribution, surface chemistry, ligand display, and biological performance across relevant cell types and tissue models (Albanese et al., 2012).

The scalability of organelle-targeted nanomaterials is limited by batch-to-batch variability, reproducibility, and cost-effectiveness. For morphology-centered studies, this variability is especially important because small changes in size, charge, or ligand density can shift intracellular trafficking and produce different structural readouts.

For MCS-directed nanomaterials, manufacturing variability may additionally alter the probability of reaching a contact interface and the magnitude of contact-site remodeling. Batch-dependent changes in particle size, surface chemistry, ligand density, deformability, or release kinetics could shift a formulation from controlled MCS modulation to lysosomal sequestration, nonspecific membrane association, or organelle stress. Reproducibility should therefore be assessed not only through conventional physicochemical characterization but also through standardized measurements of contact-site localization, architecture, and function.

Microfluidics-based synthesis and automated nanoparticle production may help address these limitations (Karnik et al., 2008). High-throughput synthesis, in-process quality control, and standardized structural assays will be crucial for ensuring that organelle-targeted nanomaterials produce reproducible localization and morphology-related outcomes.

### 7.5 Variability across cell types, tissues, and biological barriers

To efficiently study or modulate organelles, nanomaterials must navigate different cell types, tissue architectures, and biological barriers while maintaining predictable intracellular behavior (Blanco et al., 2015). Intracellular variation in membrane composition, endocytic activity, cytoskeletal organization, organelle abundance, and extracellular matrix context can alter uptake, trafficking, and organelle localization. These variables are particularly important when moving from immortalized cell lines to primary cells, stem cells, organoids, or tissue models.

MCS architecture is likewise cell-state and tissue dependent. The abundance, molecular composition, spacing, and functional role of ER–mitochondria, mitochondria–lysosome, and ER–endolysosomal contacts may vary with differentiation status, metabolic demand, disease state, and tissue organization. Consequently, a nanomaterial that alters a contact site in one cell model may produce a different structural or functional outcome in another, even when cellular uptake is comparable. MCS-directed effects should therefore be validated across physiologically relevant cell types and tissue models rather than inferred from a single immortalized cell line.

A stratified approach may be useful for matching nanomaterial design to a specific cell type, tissue environment, or disease-associated structural phenotype (Yokoi et al., 2014). Biomarkers of cell state, organelle stress, or tissue remodeling could help guide selective targeting, but such strategies require direct evidence of cell-specific and organelle-specific localization rather than generalized uptake.

### 7.6 Translational challenges and requirements for clinical progression

Translation of organelle- and MCS-directed nanomaterials requires more than demonstration of intracellular uptake or short-term efficacy in cultured cells. Major barriers include endosomal sequestration, variable protein-corona formation, off-target organelle accumulation, dose-dependent membrane injury, limited biodegradability, and differences in intracellular trafficking among cell types and tissues (Behzadi et al., 2017; Allen and Yokota, 2024). Increasing structural complexity through multiple ligands, responsive components, or sequential targeting mechanisms may enhance experimental precision but can also complicate scalable manufacturing, quality control, stability, and batch-to-batch reproducibility.

Clinical progression will therefore require standardized physicochemical characterization, quantitative confirmation of subcellular localization, physiologically relevant cellular and *in vivo* models, and longitudinal evaluation of organelle, genomic, immune, and tissue safety. For MCS-directed systems, additional evidence should demonstrate contact-site localization, modulation of a defined contact-dependent process, and reversibility of the structural effect. Consequently, highly complex multi-organelle and MCS-directed platforms are likely to face greater translational barriers than simpler biodegradable systems with reproducible manufacturing procedures and clearly defined mechanisms.

## 8 Future directions for MCS-directed and tissue-oriented intracellular nanomaterials

Organelle-targeted nanomaterials are moving toward more precise and mechanistically interpretable designs. Future studies should prioritize structural evidence of changes in organelle morphology, inter-organelle contact sites, chromatin organization, autophagy, mitophagy, and tissue-relevant behavior (Friedman et al., 2011). Because direct MCS-targeted systems remain limited and predominantly preclinical, the concepts below should be regarded as research directions rather than established applications. Progress will require materials that reach defined interfaces or regulate specific tethering, ion-transfer, lipid-exchange, organelle-division, or membrane-repair mechanisms, together with nanoscale structural, functional, and causal validation.

### 8.1 Artificial intelligence (AI)-driven design for targeting precision and morphology-aware validation

Integrating AI and machine learning into nanomaterial design is being explored as a way to optimize multi-parameter platforms for subcellular localization (Furxhi et al., 2020). For morphology-centered applications, AI models should not only predict uptake or delivery efficiency, but also infer relationships between material properties and structural readouts such as organelle size, cristae organization, nuclear morphology, lysosomal distribution, and tissue-level compatibility.

For MCS-focused applications, AI-assisted analysis may also support automated quantification of contact abundance, length, spacing, duration, subcellular distribution, and molecular composition from imaging datasets. Integrating these structural features with particle properties and functional readouts such as Ca^2+^ transfer, lipid exchange, respiration, mitophagy, or membrane repair could help identify material–contact-site relationships that are not apparent from uptake measurements alone. However, such models will require standardized annotations and independent validation across imaging platforms, cell types, and tissue models.

AI-driven and data-guided approaches may accelerate nanomaterial discovery by optimizing size, surface chemistry, geometry, and ligand density. However, progress depends on high-quality standardized datasets with consistent metadata, including cell type, tissue model, dose, time point, imaging modality, localization assay, and structural endpoint. Generalizability remains a major constraint because models trained on immortalized cell lines may not transfer to primary cells, stem cells, organoids, or *in vivo* tissues. Therefore, AI-guided candidates should be evaluated prospectively with pre-specified structural metrics and orthogonal assays of subcellular localization and function.

### 8.2 Programmable nanomaterials for dynamic organellar localization

Programmable nanomaterials that adapt to heterogeneous intracellular environments are being actively explored. Stimuli-responsive systems can change charge exposure, hydrodynamic size, ligand presentation, or cargo release in response to pH, redox status, enzyme activity, or external triggers (Ganta et al., 2008; Torchilin, 2014). For cell-structure studies, the key question is whether these changes produce verified organelle-localized action and interpretable morphological consequences rather than generalized stress. Progress will require standardized readouts that confirm staged trafficking and triggered release at intended subcellular sites, alongside dose-response and longitudinal safety profiling.

For example, instead of relying on lysosomal perturbation, programmable systems may be designed for timed endosomal escape or lysosome-avoidance routing, followed by triggered release of cargo in the cytosol and subsequent targeting to mitochondria or the nucleus depending on the cellular context.

For MCS-directed applications, programmable systems may also be designed to expose targeting motifs or release functional cargo only after reaching a defined inter-organelle interface. Potential strategies include responsiveness to local Ca^2+^, redox state, lipid composition, membrane potential, or contact-associated enzymes. However, stimulus responsiveness alone does not establish contact-site specificity; studies should demonstrate spatial activation at the intended interface and verify the corresponding change in contact architecture or contact-dependent function.

### 8.3 Gene editing and nanotechnology integration with nuclear structural validation

The integration of nanotechnology with gene-editing systems such as CRISPR/Cas9 is being explored as a route to improve intracellular delivery and enable cell-type-specific genome modulation (Mout et al., 2017). From a structural perspective, however, this area requires rigorous confirmation of nuclear delivery, editing outcomes, and nuclear integrity. Progress will require orthogonal validation of intranuclear localization where relevant, on- and off-target profiling by sequencing, and longitudinal studies of genotoxicity, immune responses, chromatin organization, and durability of edited phenotypes (Kosicki et al., 2018).

Gene-editing approaches may also provide causal tools for MCS research by selectively modifying contact-site tethers, lipid-transfer proteins, ion channels, or untethering regulators. Nanomaterial-mediated delivery of CRISPR components could therefore be used not only to alter nuclear genes, but also to test whether changes in nanomaterial-induced metabolism, organelle dynamics, or cell fate depend on specific ER–mitochondria, mitochondria–lysosome, or ER–endolysosomal machinery. Such studies should combine editing validation with quantitative structural and functional assessment of the targeted contact site.

### 8.4 Stratified approaches for tissue- and cell-type-specific organellar targeting

Stratified intracellular nanotechnology may enable organelle-targeted designs to be matched to tissue type, cell state, disease-associated structural changes, or regenerative context (Sago et al., 2018; Dilliard et al., 2021). This approach is constrained by variability in biomarker robustness across patients and disease stages, incomplete understanding of differences in intracellular trafficking and protein corona formation, and practical challenges in scalable manufacturing and quality control. Progress will require validated biomarker panels linked to organelle-relevant mechanisms and standardized assays confirming cell-type and organelle-specific localization, not merely uptake.

Stratification may also be required at the level of MCSs because their abundance, molecular composition, and functional roles differ across cell types, differentiation states, tissues, and pathological conditions. Future MCS-directed nanomaterials should therefore be matched to contact-site phenotypes, such as altered ER–mitochondria coupling, defective mitochondria–lysosome dynamics, or impaired ER–lysosome membrane repair. Candidate systems should be evaluated using cell- and tissue-specific structural and functional benchmarks rather than assuming that the same MCS-targeting strategy will produce comparable effects across biological models.

Advances in biomarker discovery, tissue modeling, organoid systems, and cell-specific targeting could enable nanomaterials to be designed around defined organelle phenotypes and tissue architectures. Such work should connect targeting strategies to observable structural endpoints, including cellular organization, organelle morphology, and histological measures of tissue repair or damage.

### 8.5 Multi-organelle targeting for cell differentiation, tissue repair, and regenerative morphology

Multi-organelle targeting is being explored in regenerative and stem-cell contexts, where coordinated modulation of bioenergetics, stress signaling, autophagy, and gene regulatory programs may support tissue repair. For example, combined mitochondrial targeting to influence bioenergetic state alongside nuclear delivery of defined regulators could, in principle, promote regenerative programs in selected contexts. However, this strategy is constrained by the difficulty of verifying simultaneous organelle specificity in tissue models, the risk of stress-mediated or off-target effects that confound regeneration claims, and uncertainty about long-term genomic and epigenomic stability. Progress will require orthogonal confirmation of organelle-localized action, standardized morphological and histological readouts of regeneration, and longitudinal assessment of durability, immune responses, and safety.

Future regenerative strategies should distinguish simultaneous organelle targeting from deliberate modulation of the contact sites that coordinate these organelles. MCS-directed systems could, in principle, regulate ER–mitochondria Ca^2+^ and metabolic coupling, mitochondria–lysosome quality control, or ER–lysosome membrane repair to influence differentiation and tissue recovery. However, regenerative benefits should be attributed to MCS regulation only when changes in a defined contact interface are demonstrated together with contact-dependent functional outcomes and durable tissue-level improvement.

## 9 Conclusion

MCSs provide a mechanistic framework for understanding how intracellular nanomaterials influence coordinated organelle behavior. By organizing localized ion and lipid exchange, organelle division, metabolic adaptation, membrane repair, and stress signaling, these interfaces connect nanoscale structural changes with cell-fate and tissue-level outcomes. Organelle localization or multi-organelle responses alone, however, do not establish direct MCS modulation.

This review integrates MCS biology with the intracellular processes that govern nanomaterial uptake, endosomal escape, organelle sorting, mitochondrial and nuclear responses, lysosomal quality control, and multi-organelle signaling. A central distinction is made among intentional organelle regulation, indirect MCS-associated effects arising from organelle perturbation, and direct contact-site engagement supported by structural and functional evidence.

Intracellular nanotechnology is most relevant to cell and tissue biology when it is framed around the organization, morphology, and functional coupling of cells, subcellular compartments, and tissues. This review outlines how organelle-targeted nanomaterials can be used to interrogate and modulate interconnected pathways linking mitochondria, lysosomes, and the nucleus, with emphasis on structural endpoints such as mitochondrial morphology, endolysosomal trafficking, mitophagy, nuclear access, chromatin organization, and inter-organelle communication.

Mitochondrial, nuclear, and lysosomal targeting should therefore be interpreted as a framework for studying cellular architecture and tissue-relevant cell fate rather than simply as a carrier strategy. Coordinated modulation of these organelles may influence differentiation, survival, apoptosis, stress adaptation, and regenerative behavior, but these outcomes should be supported by morphological, ultrastructural, histological, and functional evidence.

Current evidence for direct nano–MCS targeting remains limited. Future studies should combine high-resolution or ultrastructural localization with quantitative assessment of contact abundance, spacing, duration, molecular composition, and the relevant Ca^2+^, lipid, metabolic, or stress-signaling process. Perturbation or rescue of contact-site machinery will be important for establishing causality and separating controlled regulation from oxidative, lysosomal, mitochondrial, inflammatory, or genotoxic injury.

Progress is constrained by endolysosomal sequestration, limited organelle and contact-site specificity, manufacturing variability, off-target structural effects, and uncertainty regarding long-term genomic, epigenomic, organelle, immune, and tissue safety. AI-guided design, programmable materials, gene-editing tools, and cell- or tissue-stratified approaches may help address these barriers, but translation will depend on reproducible manufacturing, physiologically relevant models, and longitudinal safety. A morphology-centered framework that integrates contact-site architecture with contact-dependent function offers the clearest route toward mechanistically precise intracellular nanotechnology.

## Review methodology

This article was prepared as a narrative review focused on organelle-targeted nanomaterials and their roles in intracellular organization, mitochondrial–nuclear communication, lysosomal and endolysosomal trafficking, organelle morphology, and cell fate regulation. Relevant literature was identified through searches of major scientific databases, including PubMed, Scopus, Web of Science, and Google Scholar. Search terms included combinations of “membrane contact sites,” “organelle contact sites,” “ER–mitochondria contacts,” “mitochondria–lysosome contacts,” “ER–endosome contacts,” “ER–lysosome contacts,” “contact-site tethers,” “intermembrane spacing,” “organelle-targeted nanomaterials,” “mitochondrial targeting,” “nuclear targeting,” “lysosomal trafficking,” “endosomal escape,” “mitochondria–nucleus crosstalk,” “nanomaterials and cell fate,” “organelle morphology,” “mitophagy,” and “intracellular nanoregulation.” Priority was given to peer-reviewed original research articles, mechanistic studies, and recent reviews that provided evidence on nanomaterial localization, intracellular trafficking, organelle-specific effects, ultrastructural changes, and functional cellular outcomes. Studies were included when they were directly relevant to the review’s focus on intracellular compartmentalization, organelle structure-function relationships, and tissue-relevant cell behavior. Articles with limited mechanistic relevance, insufficient connection to organelle-level regulation, or primarily descriptive delivery outcomes without structural or functional validation were not emphasized. The selected literature was synthesized qualitatively to develop a morphology-centered framework for evaluating intracellular nanomaterials in cell and tissue organization.
